# An Enhanced Progressive Damage Model for Laminated Fiber-Reinforced Composites Using the 3D Hashin Failure Criterion: A Multi-Level Analysis and Validation

**DOI:** 10.3390/ma17215176

**Published:** 2024-10-24

**Authors:** Yichen Zhang, Wim Van Paepegem, Wouter De Corte

**Affiliations:** 1 Department of Structural Engineering and Building Materials, Faculty of Engineering and Architecture, Ghent University, Tech Lane Ghent Science Park 60, 9052 Zwijnaarde, Belgium; yichen.zhang@ugent.be; 2 Department of Materials, Textiles and Chemical Engineering, Faculty of Engineering and Architecture, Ghent University, Tech Lane Ghent Science Park 46, 9052 Zwijnaarde, Belgium; wim.vanpaepegem@ugent.be

**Keywords:** FRP, progressive damage model (PDM), multi-level analysis, non-monotonic loading, characteristic length, numerical simulation

## Abstract

This paper presents a progressive damage model (PDM) based on the 3D Hashin failure criterion within the ABAQUS/Explicit^TM^ 2021 framework via a VUMAT subroutine, enhancing the characterization of the mechanical performance and damage evolution in the elastic and softening stages of composite materials via the accurate calculation of damage variables and accommodation of non-monotonic loading conditions. In the subsequent multi-level verification, it is found that the model accurately simulates the primary failure modes at the element level and diminishes the influence of element size, ensuring a reliable behavior representation under non-monotonic loading. At the laminate level, it also accurately forecasts the elastic behavior and damage evolution in open-hole lamina and laminates, demonstrating the final crack band at ultimate failure. This paper also emphasizes the importance of correct characteristic length selection and how to minimize mesh size impact by selecting appropriate values. Compared to ABAQUS’s built-in 2D model, the 3D VUMAT subroutine shows superior accuracy and effectiveness, proving its value in characterizing the mechanical behavior and damage mechanisms of fiber-reinforced polymer (FRP) materials. The enhanced 3D PDM accurately characterizes the softening processes in composite materials under simple or complex stress states during monotonic or non-monotonic loading, effectively minimizes the mesh dependency, and reasonably captures failure crack bands, making it suitable for future simulations and resolutions of numerical issues in composite material models under complex, three-dimensional stress states.

## 1. Introduction

In the field of material engineering, fiber-reinforced polymers (FRPs) stand out as lightweight materials for modern structural applications, offering advantages in terms of strength-to-weight ratios, corrosion resistance, and design flexibility [1]. These composite materials, typically composed of a polymer matrix reinforced with fibers such as glass, carbon, or aramid [2], have revolutionized industries ranging from aerospace, automotive, and medical to construction and sports equipment [3,4,5,6,7]. The unique properties of FRPs allow for the creation of components that are both lightweight and exceptionally strong, making them indispensable in high-performance applications [8].

Simulating the behavior of FRPs under various stress states is crucial for enhancing their performance and ensuring reliability in practical applications [9,10]. Among the numerous theories for predicting the initiation of damage in Unidirectional Fiber Reinforced Plastic (UD-FRP) laminates, the Hashin [11], Puck [12], and LaRC05 [13] failure criteria are widely recognized in the industry due to their high precision results. The Hashin failure criterion stands as one of the most mature frameworks for understanding and predicting the failure behavior of unidirectional composite materials. Its practicality and effectiveness have been extensively validated in industrial applications. The criterion offers a robust methodology for assessing different failure modes in the fiber and matrix components of composites, thereby facilitating a comprehensive analysis of mechanical failures under diverse loading conditions. Concurrently, progressive damage models (PDMs) [14,15,16] play a pivotal role in the failure analysis of FRP composite components. By linking the degradation of material stiffness with the micro-damage mechanisms in fibers, matrices, and interfaces, these models effectively predict the onset of damage and its progression under complex loading conditions [14,17,18,19,20,21,22,23,24].

For practical applications, a 2D PDM based on the Hashin failure criterion has been incorporated into the Abaqus/Explicit^TM^ framework. Additionally, numerous researchers have developed PDMs that do not consider stress–strain components in the thickness direction. Maimi et al. [25] proposed a continuum damage model based on the LaRC04 failure criteria designed to predict the mechanical behavior of composite laminates under a plane stress state. Fakoor et al. [26] conducted a sensitivity analysis on their established two-dimensional damage model concerning the stiffness degradation methods associated with material softening. Ridha et al. [27] and Su et al. [28] used their developed 2D PDM to predict the strength and failure modes of notched laminates. However, despite the extensive application of the standard 2D Hashin model available from the ABAQUS material library, in this study, further referred to as the 2D Hashin model, its use in three-dimensional contexts such as impact, indentation, and drilling has been relatively limited.

To address this issue, several three-dimensional progressive damage models (PDMs) have been developed. Donadon et al. [20] and Li et al. [29] employed these models to predict the mechanical response of composite laminates subjected to low-velocity impact damage, although matrix failure in the thickness direction was not included in their analysis. Huang et al. [19] and Xin et al. [21] utilized developed three-dimensional damage models to predict the mechanical responses of FRP composites under impact loading. Additionally, Divse et al. [16] focused on modeling the mechanical behavior of laminates, which included drilling-induced damage. While Maimi et al. [30] proposed a model that accounts for stiffness degradation in the thickness direction, it neglects the degradation of the shear modulus G_23_. Finally, Lee et al. [31] employed a three-dimensional model to simulate tensile tests on open-hole laminates.

In summary, the use of three-dimensional models to characterize the mechanical behavior of FRP composites has generally achieved relatively accurate results. However, these studies generally calculate the equivalent damage initial strain by dividing the ultimate strength by the elastic modulus directly [14,16,25,29,32,33,34]. Under complex stress conditions, this approach is not accurate. Moreover, there is an absence of literature on three-dimensional progressive damage models that concurrently consider the degradation of out-of-plane stiffness and non-monotonic loading while also providing a comprehensive, multi-level validation.

To address this deficiency, this paper presents a progressive damage model (PDM) that incorporates the 3D Hashin failure criterion within the ABAQUS VUMAT framework. This model explicitly considers stiffness degradation both in the thickness direction and resulting from non-monotonic loading. Furthermore, the innovative model significantly improves the predictive accuracy of finite element simulations by introducing a novel methodology for determining equivalent initial damage strain and stress. Subsequently, the model undergoes thorough validation across various scales, from the element level to the laminate level. The findings confirm that the model is capable of accurately predicting the mechanical behavior of composites under diverse and complex stress states, including non-monotonic conditions.

## 2. Continuum Damage Framework Based on 3D Hashin Criterion

This section provides a detailed exposition of the theoretical foundation and implementation process of the developed PDM based on the 3D Hashin failure criterion. A notable innovation of this model lies in the adoption of a novel approach for determining the equivalent damage initiation strain $\varepsilon_{eq}^{0,i}$ and stress $\sigma_{eq}^{0,ft}$, as will be discussed in Section 2.3. This method enables a more accurate calculation of the equivalent damage initiation parameters under complex stress states, thereby more precisely characterizing the initiation and evolution of Hashin damage. Additionally, the PDM incorporates degradation of stiffness in the thickness direction and the effects of non-monotonic loading while utilizing the characteristic length of elements to reduce the dependency of the simulation results on the mesh size. Section 2 elaborates on the theoretical underpinnings of the model as well as the methodology for determining key parameters in the subroutine. Finally, a flowchart is presented to illustrate the complete process of damage evolution at material points as implemented in ABAQUS/Explicit^TM^ via a VUMAT subroutine.

### 2.1. Stiffness Formulation

In the presence of damage, the stiffness matrix of a unidirectional laminate (UD) undergoes corresponding changes. In this study, the damage stiffness matrix proposed by Kachanov [22] and Matzenmiller et al. [23] for plane stress conditions was extended to three-dimensional stress states [15]. The relationship between the damage tensor M, the effective stress $\tilde{\sigma }$, and the nominal stress σ is depicted in Equation (1), whereby M is a diagonal matrix composed of damage parameters, as shown in Equation (2):

(1) $$\begin{array}{c} \tilde{\sigma }=M\sigma , \end{array}$$

(2) $$M=\left[\begin{array}{cccccc} \frac{1}{1-d_{f}} & 0 & 0 & 0 & 0 & 0\\ 0 & \frac{1}{1-d_{m}} & 0 & 0 & 0 & 0\\ 0 & 0 & \frac{1}{1-d_{m}} & 0 & 0 & 0\\ 0 & 0 & 0 & \frac{1}{1-d_{s}} & 0 & 0\\ 0 & 0 & 0 & 0 & \frac{1}{{1-d}_{s}} & 0\\ 0 & 0 & 0 & 0 & 0 & \frac{1}{{1-d}_{s}} \end{array}\right]$$

Here, the variables $d_{f}$, $d_{m}$ and $d_{s}$ (ranging from 0 to 1), respectively, represent global damage variables for fiber, matrix, and shear modes. They can be expressed using the following Equation (3):

(3) $$\begin{array}{c} \left\{\begin{array}{c} d_{f}=1-\left(1-d_{ft}\right)\left(1-d_{fc}\right)\\ d_{m}=1-\left(1-d_{mt}\right)\left(1-d_{mc}\right)\\ d_{s}=1-\left(1-S_{mt}d_{mt}\right)\left(1-S_{mc}d_{mc}\right) \end{array}\right. \end{array}$$

In the above equations, $d_{ft}$, $d_{fc}$, $d_{mt}$, and $d_{mc}$ (ranging from 0 to 1) represent local damage variables for fiber compression, fiber tension, matrix compression, and matrix tension, respectively, which are determined using damage evolution methodologies. When the value of these variables reaches 1, it indicates that the components in that specific direction are completely damaged. The calculation methods for these four values will be discussed in Section 2.2. Additionally, to control the shear stiffness degradation due to matrix failure in tension and compression, the coefficients $S_{mt}$ and $S_{mc}$ are introduced, with assumed values of 0.9 and 0.5, respectively [31,32,35,36].

Subsequently, the compliance matrix of the damaged laminate is obtained through Equation (4), while Equation (5) represents the compliance matrix $S_{d}$ of the material after damage.

(4) $$\begin{array}{c} S_{d}=SM \end{array}$$

(5) $$S_{d}=\left[\begin{array}{cccccc} \frac{1}{{(1-d}_{f})E_{11}} & \frac{-\nu_{21}}{E_{22}} & \frac{-\nu_{31}}{E_{33}} & 0 & 0 & 0\\ \frac{-\nu_{12}}{E_{11}} & \frac{1}{(1-d_{m})E_{22}} & \frac{-\nu_{32}}{E_{33}} & 0 & 0 & 0\\ \frac{-\nu_{13}}{E_{11}} & \frac{-\nu_{23}}{E_{22}} & \frac{1}{(1-d_{m})E_{33}} & 0 & 0 & 0\\ 0 & 0 & 0 & \frac{1}{(1-d_{s})G_{12}} & 0 & 0\\ 0 & 0 & 0 & 0 & \frac{1}{{(1-d}_{s})G_{23}} & 0\\ 0 & 0 & 0 & 0 & 0 & \frac{1}{{(1-d}_{s})G_{31}} \end{array}\right]$$

Further, the damaged stiffness matrix $C_{d}$ is obtained by calculating the inverse of the compliance matrix $S_{d}$ affected by damage. The obtained damaged stiffness matrix $C_{d}$ is applied in the VUMAT developed in this study, and stress updates are performed using Equation (7).

(6) $$C_{d}={S_{d}}^{-1}$$

(7) $$\sigma =C_{d}\varepsilon$$

(8) $$C_{d}=\left[\begin{array}{cccccc} {dC}_{11} & {dC}_{12} & {dC}_{13} & 0 & 0 & 0\\ {dC}_{12} & dC_{22} & {dC}_{23} & 0 & 0 & 0\\ {dC}_{13} & {dC}_{23} & dC_{33} & 0 & 0 & 0\\ 0 & 0 & 0 & {dC}_{44} & 0 & 0\\ 0 & 0 & 0 & 0 & {dC}_{55} & 0\\ 0 & 0 & 0 & 0 & 0 & {dC}_{66} \end{array}\right]$$

(9) $$\begin{array}{c} {dC}_{11}=(1-d_{f})E_{11}\left[1-{\left(1-d_{m}\right)}^{2}v_{23}v_{32}\right]/∆\\ {dC}_{22}={(1-d}_{m})E_{22}\left[1-{(1-d}_{f})(1-d_{m})v_{13}v_{31}\right]/∆\\ {dC}_{33}={(1-d}_{m})E_{33}\left[1-{(1-d}_{f})(1-d_{m})v_{12}v_{21}\right]/∆\\ {dC}_{12}=(1-d_{f})(1-d_{m})E_{11}\left[(1-d_{m})v_{31}v_{23}+v_{21}\right]/∆\\ {dC}_{13}={(1-d}_{f})({1-d}_{m})E_{11}\left[{(1-d}_{m})v_{21}v_{32}+v_{31}\right]/∆\\ {dC}_{23}={\left({1-d}_{m}\right)}^{2}E_{22}\left[{(1-d}_{f})v_{12}v_{31}+v_{32}\right]/∆\\ {dC}_{44}={(1-d}_{s})G_{12}\\ {dC}_{55}={(1-d}_{s})G_{23}\\ {dC}_{66}={(1-d}_{s})G_{31} \end{array}$$

where

(10) $$\begin{array}{c} \Delta =1-{\left(1-d_{m}\right)}^{2}v_{32}v_{23}-{(1-d}_{f})(1-d_{m})v_{31}v_{13}-(1-d_{f})(1-d_{m})v_{21}v_{12}\\ -2({1-d}_{f}){\left(1-d_{m}\right)}^{2}v_{13}v_{21}v_{32} \end{array}$$

### 2.2. Damage Initiation Criteria

The failure criteria for unidirectional composite materials are typically represented by stress polynomial functions related to the modes of failure. To account for the damage initiation state of UD (unidirectional) lamina under three-dimensional stress conditions, the 3D Hashin criteria [11] were incorporated into this work. This progressive damage initiation criterion considers four types of damage modes: fiber tension (FT), fiber compression (FC), matrix tension (MT), and matrix compression (MC). By developing a user-defined material subroutine (VUMAT), these four three-dimensional failure criteria are implemented in ABAQUS.

The three-dimensional Hashin damage initiation criteria [11] are expressed mathematically as Equations (11)–(14).

(1) The fiber tension mode ($\sigma_{11}\geq 0$):
  (11) $$\begin{array}{c} F_{ft}={\left(\frac{\sigma_{11}}{X_{T}}\right)}^{2}+{\alpha \left(\frac{\sigma_{12}}{S_{12}}\right)}^{2}+{\alpha \left(\frac{\sigma_{13}}{S_{13}}\right)}^{2}\left(\alpha =1\right) \end{array}$$
(2) The fiber compression mode ($\sigma_{11}\leq 0$):
  (12) $$\begin{array}{c} F_{fc}=\frac{-\sigma_{11}}{X_{C}} \end{array}$$
(3) The matrix tension mode ($\sigma_{22}+\sigma_{33}\geq 0$):
  (13) $$\begin{array}{c} F_{mt}={\left(\frac{\sigma_{22}+\sigma_{33}}{Y_{T}}\right)}^{2}+\frac{\sigma_{23}^{2}-\sigma_{22}\sigma_{33}}{S_{23}^{2}}+{\left(\frac{\sigma_{12}}{S_{12}}\right)}^{2}+{\left(\frac{\sigma_{13}}{S_{13}}\right)}^{2} \end{array}$$
(4) The matrix compression mode ($\sigma_{22}+\sigma_{33}\leq 0$):
  (14) $$\begin{array}{c} F_{mc}=\frac{1}{Y_{C}}\left({\left(\frac{Y_{C}}{2S_{23}}\right)}^{2}-1\right)\left(\sigma_{22}+\sigma_{33}\right)+\frac{{\left(\sigma_{22}+\sigma_{33}\right)}^{2}}{4S_{23}^{2}}+\frac{\sigma_{23}^{2}-\sigma_{22}\sigma_{33}}{S_{23}^{2}}+{\left(\frac{\sigma_{12}}{S_{12}}\right)}^{2}+{\left(\frac{\sigma_{13}}{S_{13}}\right)}^{2} \end{array}$$

$F_{ft}$, $F_{fc}$, $F_{mt}$, and $F_{mc}$ represent the damage initiation parameters for each mode, respectively. $\sigma_{ij}$ denotes the stress components in respective directions. $X_{T}$ and $X_{C}$ denote the longitudinal tensile and compressive strengths of the UD lamina, while $Y_{T}$ and $Y_{C}$ denote the transverse tensile and compressive strengths. $S_{12}$, $S_{23}$, and $S_{13}$ denote the shear strengths in the three respective directions. The ultimate strengths for all failure modes mentioned and incorporated into the Hashin criterion are positive. Each damage initiation parameter ranges from 0 to 1. When any damage initiation parameter reaches 1, it indicates that damage begins to evolve in the corresponding mode.

### 2.3. Damage Evolution

It is crucial to determine the mechanical behavior of UD-FRP lamina after damage. Prior to any damage, the material exhibits linear elastic behavior. Therefore, the stiffness matrix before damage can be determined by following the constitutive relationship given by Equation (8), assuming the global damage variables ($d_{f}$, $d_{m}$, and $d_{s}$) are set to 0. Since further loading after damage occurs results in a decrease in the stiffness of the laminated structure, the stiffness matrix (Equation (8)) is updated accordingly, whereby the global damage variables start increasing from 0. These global damage variables are computed using four local damage variables $d_{i}$ (*i* = *ft, fc, mt, mc*), as shown in Equation (3), with their respective values increasing from 0 to 1. To evaluate the local damage variables $d_{i}$, Equation (15) is employed.

It should be noted that both global and local damage variables are scaled from 0 to 1; a value of 0 means no damage, and a value of 1 means completely damaged.

In Equation (15), $\varepsilon_{eq}^{i}$ represents the equivalent strain for a certain damage mode, and its calculation is detailed in Equations (18)–(21). Additionally, the equivalent displacement $\delta_{eq}^{i}$ and equivalent stress $\sigma_{eq}^{i}$ of the element can be obtained through the equivalent strain $\varepsilon_{eq}^{i}$ and the characteristic length $L_{c}$ (see Table 1).

In Equation (15), $\varepsilon_{eq}^{0,i}$ and $\varepsilon_{eq}^{f,i}$, respectively, correspond to the equivalent strains at the onset of damage and at the final damage for each mode. The superscripts indicate the four damage modes: fiber tension (FT), fiber compression (FC), matrix tension (MT), and matrix compression (MC). Accurately determining these parameters, $\varepsilon_{eq}^{0,i}$ and $\varepsilon_{eq}^{f,i}$, is crucial for correctly describing the mechanical behavior during the stiffness-degradation phase. It is important to clarify that when the damage initiation variable $F_{i}=1$, the equivalent strain $\varepsilon_{eq}^{i}$ at the onset of damage for the material point under that damage mode is represented as $\varepsilon_{eq}^{0,mt}$.

In all the literature reviewed to date [14,16,25,29,32,33,34], $\varepsilon_{eq}^{0,i}$ is determined by Equation (16), and the initial strain of damage obtained by this method is accurate when one of the normal or shear components is not significant. However, when both normal and shear components cannot be neglected, the calculated equivalent initial strain $\varepsilon_{eq}^{o,i}$ is not as accurate. This discrepancy arises when an element is in a complex stress state, where $\varepsilon_{eq}^{0,i}$ should not be solely calculated by Equation (16) while neglecting shear components.

For instance, in the case of a laminate containing ±45° plies under tension, the in-plane shear stress, $\sigma_{12}$, significantly influences the material’s damage initiation parameter, $F_{mt}$. If Equation (16) is used to calculate the initial damage strain ($\varepsilon_{eq}^{0,mt}$), it completely ignores the contribution of the shear component to the $\varepsilon_{eq}^{0,mt}$, which is clearly unreasonable.

Therefore, this paper adopts a new method to determine $\varepsilon_{eq}^{0,i}$ of an element in each damage mode, which no longer pre-calculates the equivalent initial damage strain using fixed material ultimate strengths and elastic moduli. Instead, it strictly follows the Hashin failure criterion, determining the equivalent strain at the moment the damage initiation variable $F_{i}$ first equals 1 for the respective damage mode, and uses this value as the initial equivalent strain for the element. Using this method, the damage-initiation stage and the stiffness-degradation stage are connected, which ensures that the initial equivalent strain $\varepsilon_{eq}^{0,i}$ can be obtained for each element depending on its real stress state.

Similarly, the equivalent final strain $\varepsilon_{eq}^{f,i}$ is no longer calculated using Equation (17). Instead, when the damage initiation variable $F_{i}$ equals to 1 for the first time, the equivalent initial damage stress $\sigma_{eq}^{o,i}$ is calculated using the equations in Table 1. Subsequently, the equivalent final damage strain $\varepsilon_{eq}^{f,i}$ in this stress state is calculated by selecting the corresponding equations from Equations (18)–(21) according to the damage mode in which the element is located. Equations (18)–(21) include the fracture energy for each failure mode $G_{c}^{i}$ and characteristic length $L_{c}$.

**Figure 1:**
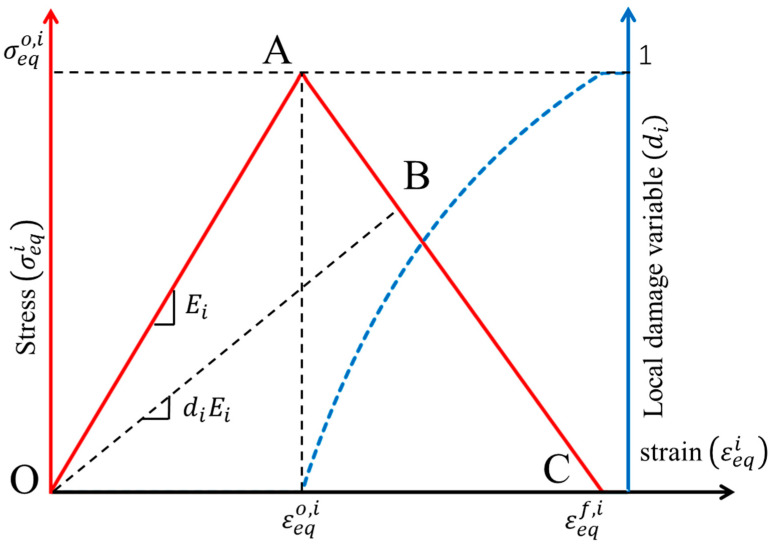
Stress–strain response of ideal linear softening model in this PDM.

The local stiffness degradation adopted in this paper follows a linear softening model, as illustrated in Figure 1. This model effectively captures the progressive reduction in stiffness as damage evolves, providing a more accurate and realistic simulation of the material behavior under various loading conditions.

(15) $$\begin{array}{c} d_{i}=\frac{\varepsilon_{eq}^{f,i}\left(\varepsilon_{eq}^{i}-\varepsilon_{eq}^{o,i}\right)}{\varepsilon_{eq}^{i}\left(\varepsilon_{eq}^{f,i}-\varepsilon_{eq}^{o,i}\right)}\left(i=ft,fc,mt,mc\right) \end{array}$$

(16) $$\varepsilon_{eq}^{0,i}=\frac{X_{i}}{E_{i}}$$

(17) $$\varepsilon_{eq}^{f,i}=\frac{2G_{c}^{i}}{X_{i}L_{c}}$$

(1) The tensile fiber mode ($\sigma_{11}\geq 0$):
  (18) $$\begin{array}{c} \varepsilon_{eq}^{ft}=\sqrt{{\left\langle \varepsilon_{11}\right\rangle }^{2}+{\varepsilon_{12}}^{2}+{\varepsilon_{13}}^{2}}\\ \varepsilon_{eq}^{0,ft}={\left.\varepsilon_{eq}^{ft}\right|}_{F_{ft}=1}\\ \sigma_{eq}^{0,ft}={\left.\sigma_{eq}^{ft}\right|}_{F_{ft}=1}\\ \begin{array}{c} \varepsilon_{eq}^{f,ft}=\frac{2G_{c}^{ft}}{\sigma_{eq}^{0,ft}L_{c}} \end{array} \end{array}$$
(2) The compressive fiber mode ($\sigma_{11}\leq 0$):
  (19) $$\begin{array}{c} \varepsilon_{eq}^{fc}=\left\langle -\varepsilon_{11}\right\rangle \\ \varepsilon_{eq}^{0,fc}={\left.\varepsilon_{eq}^{fc}\right|}_{F_{fc}=1}\\ \sigma_{eq}^{0,fc}={\left.\sigma_{eq}^{fc}\right|}_{F_{fc}=1}\\ \begin{array}{c} \varepsilon_{eq}^{f,fc}=\frac{2G_{c}^{fc}}{\sigma_{eq}^{0,fc}L_{c}} \end{array} \end{array}$$
(3) The tensile matrix mode ($\sigma_{22}+\sigma_{33}\geq 0$):
  (20) $$\begin{array}{c} \varepsilon_{eq}^{mt}=\sqrt{{\left\langle \varepsilon_{22}\right\rangle }^{2}+{\left\langle \varepsilon_{33}\right\rangle }^{2}+{\varepsilon_{12}}^{2}+{\varepsilon_{23}}^{2}+{\varepsilon_{13}}^{2}}\\ \varepsilon_{eq}^{0,mt}={\left.\varepsilon_{eq}^{mt}\right|}_{F_{mt}=1}\\ \sigma_{eq}^{0,mt}={\left.\sigma_{eq}^{mt}\right|}_{F_{mt}=1}\\ \begin{array}{c} \varepsilon_{eq}^{f,mt}=\frac{2G_{c}^{mt}}{\sigma_{eq}^{0,mt}L_{c}} \end{array} \end{array}$$
(4) The compressive matrix mode ($\sigma_{22}+\sigma_{33}\leq 0$):
  (21) $$\begin{array}{c} \varepsilon_{eq}^{mc}=\sqrt{{\left\langle -\varepsilon_{22}\right\rangle }^{2}+{\left\langle -\varepsilon_{33}\right\rangle }^{2}+{\varepsilon_{12}}^{2}+{\varepsilon_{23}}^{2}+{\varepsilon_{13}}^{2}}\\ \varepsilon_{eq}^{0,mc}={\left.\varepsilon_{eq}^{mc}\right|}_{F_{mc}=1}\\ \sigma_{eq}^{0,mc}={\left.\sigma_{eq}^{mc}\right|}_{F_{mc}=1}\\ \begin{array}{c} \varepsilon_{eq}^{f,mc}=\frac{2G_{c}^{mc}}{\sigma_{eq}^{0,mc}L_{c}} \end{array} \end{array}$$
  where ⟨⟩ is the Macaulay bracket operator, defined as ⟨*x*⟩ = (*x* + |*x*|/2).

### 2.4. Characteristic Length

Strain softening leads to significant mesh dependency arising from strain localization, resulting in a reduction in the dissipated energy as the mesh undergoes refinement [16]. This indicates that the energy dissipated during fracture is directly proportional to the actual size of the element [37]. Consequently, the introduction of a characteristic length $L_{c}$ controls the damage process, with an accurate description of strain softening. To minimize mesh dependency during the damage process as much as possible, it is necessary to normalize the fracture energy of an element based on its characteristic length $L_{c}$.

In ABAQUS, for solid elements, the default $L_{c}$ is the cube root of the element volume [35]. In the softening model implemented in this study, the $L_{c}$ in the thickness direction of the element is equal to the actual thickness, while in the in-plane directions, it is set to the square root of the area. This configuration aims to minimize computational effort while eliminating errors in the characteristic length of thinner hexahedral elements in the in-plane directions as much as possible.

Furthermore, since the elastic energy of an element at the onset of localization must exceed its fracture energy, the damage initiation strain must be less than the final damage strain [25]. This ensures that the elements have sufficient elastic energy to drive the process before reaching localization and further softening.

(22) $$\begin{array}{c} \frac{X_{i}}{E_{i}}\leq \frac{{2G}_{ic}}{X_{i}L_{c}} \end{array}$$

Therefore, the maximum value of the $L_{c}$ is

(23) $$\begin{array}{c} L_{c}\leq \frac{2E_{i}G_{ic}}{X_{i}^{2}} \end{array}$$

This approach enhances the accuracy and reliability of the computational model, ensuring practical and precise simulation outcomes. The effect of the characteristic length on the mechanical behavior of the softening phase will be specifically analyzed in Section 4.2.

### 2.5. Implementation of 3D Hashin Progressive Damage Model

According to the 3D Hashin damage criterion described earlier in this section and based on the fracture energy, the progressive damage model was successfully implemented in the framework of Abaqus/Explicit by means of the VUMAT user subroutine. The operational logic of the subroutine is briefly described as follows:

Initially, the subroutine retrieves material properties and “old” solution-dependent variables (SDV) from the previous increment for each material point (integration point) from the finite element (FE) model. It then updates the strain of the elements based on the prescribed displacement increment. Subsequently, the local damage variable $d_{i}$ at each material point is examined. If $d_{i}=0$, indicating no degradation, stresses are computed using the elastic stiffness matrix C. Otherwise, stresses are updated based on the damaged stiffness matrix $C_{d}$ (Equation (8)).

Next, the subroutine checks whether the damage initiation variable $F_{i}^{old}$ from the last increment equals 1.

If $F_{i}^{old}=1$, this indicates that material stiffness degradation has occurred. The subroutine then determines if the current equivalent strain $\varepsilon_{eq}^{i}$ exceeds the historical maximum equivalent strain $\varepsilon_{eq}^{max,i}$. If $\varepsilon_{eq}^{i}\geq \varepsilon_{eq}^{max,i}$, the new local damage variable $d_{i}$ is computed, and $\varepsilon_{eq}^{max,i}$ is updated to $\varepsilon_{eq}^{i}$. This step of checking the $\varepsilon_{eq}^{max,i}$ is crucial for addressing scenarios where the equivalent strain $\varepsilon_{eq}^{i}$ decreases under non-monotonic loading conditions.

If $F_{i}^{old}<1$, indicating that the material point has not yet initiated damage, the subroutine calculates the Hashin damage initiation parameter $F_{i}$(i = ft, fc, mt, mc) for each failure mode according to the 3D Hashin theory (Equations (11)–(14)). If $F_{i}=1$ for the first time, it assigns $d_{i}=0$; the equivalent strain $\varepsilon_{eq}^{i}$, equivalent stress $\sigma_{eq}^{i}$, equivalent initial strain $\varepsilon_{eq}^{o,i}$, and equivalent final damage strain $\varepsilon_{eq}^{f,i}$ are calculated according to Equations (18)–(21) and Table 1; and it updates $\varepsilon_{eq}^{max,i}=\varepsilon_{eq}^{i}$.

**Figure 2:**
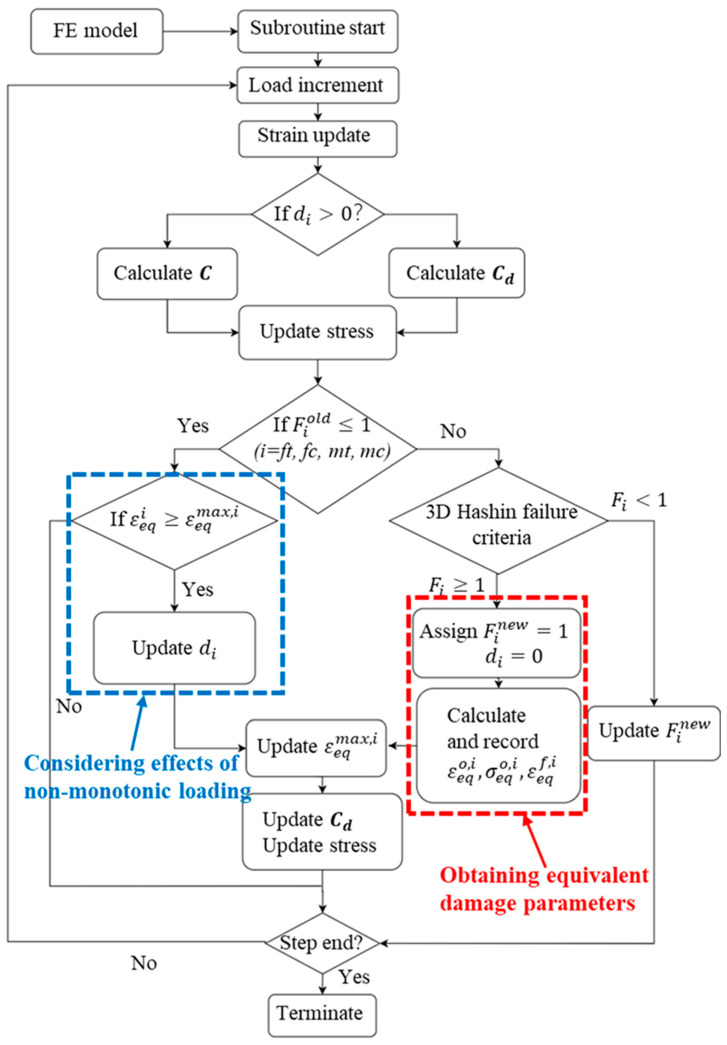
Flowchart for implementation of the PDM based on 3D Hashin criteria.

Finally, based on the newly computed local damage variable $d_{i}$, the damage stiffness matrix $C_{d}$ and stress state of the material point are updated. The entire process logic is illustrated in Figure 2.

Through this implementation, the VUMAT in this work not only accurately characterizes the damage-initiation and degradation processes at any stress state of the material point but also considers stiffness degradation in the thickness direction of the element and can be applied to non-monotonic loading conditions. Comprehensive validation of various aspects of this VUMAT subroutine will be conducted in the next section.

### 2.6. Modeling of Interlaminar Delamination

In this study, the cohesive zone model (CZM) integrated with a general contact algorithm in the ABAQUS framework is employed to simulate the interlaminar delamination in FRP laminates. The model utilizes surface-based cohesive interactions to describe the interfacial adhesive contact stress behavior through a bilinear traction–separation relationship [38,39]. We adopt the quadratic traction damage initiation criterion (Equation (24)) to predict the onset of interlaminar damage. In the equation, $t_{n}$, $t_{s}$, and $t_{t}$ represent the contact stresses in the normal and two shear directions, respectively, while $t_{n}^{\prime }$, $t_{s}^{\prime }$, and $t_{t}^{\prime }$ are the critical contact stresses in the respective modes. For the propagation of delamination, the B-K criterion [40], which is based on the mixed-mode energy release rate, is selected to characterize interface separation.

(24) $$\begin{array}{c} {\left(\frac{\left\langle t_{n}\right\rangle }{t_{n}^{\prime }}\right)}^{2}+{\left(\frac{t_{s}}{t_{s}^{\prime }}\right)}^{2}+{\left(\frac{t_{t}}{t_{t}^{\prime }}\right)}^{2}=1 \end{array}$$

## 3. Numerical Simulation Verification

The objective of this section is to validate the accuracy and general applicability of the established PDM based on the 3D Hashin failure criterion. The validation approach progresses from simple (element level) to complex (laminate level) scenarios. It should be noted that the 2D Hashin criterion in ABAQUS’s material library can only be applied to shell elements (S8R) or continuum shell elements (SC8R). Therefore, at the element level, the accuracy and superiority of the proposed 3D VUMAT are demonstrated by comparing its results with those obtained from the built-in 2D damage model in ABAQUS. At the laminate level, the accuracy of the subroutine is further corroborated by comparing the simulation results with experimental data reported in the literature [41]. All solid elements utilized in this study are eight-node linear bricks with reduced integration and hourglass control (C3D8R), while the 2D models employ eight-node, quadrilateral, in-plane, general-purpose continuum shell elements, also with reduced integration and hourglass control (SC8R).

### 3.1. Element Level

The purpose of this part is to examine whether this developed 3D VUMAT subroutine can achieve the expected mechanical responses at the element level, as specified by the inputs. The object of the study is a 3D solid element (C3D8R) with a 1 mm side length made of E-glass/Polyester. The material properties of the E-glass/Polyester are assumed (detailed in Table 2). As a comparison, a 2D element (SC8R) with the same dimensions and material parameters is also created.

#### 3.1.1. Single-Element Verification

**Figure 3:**
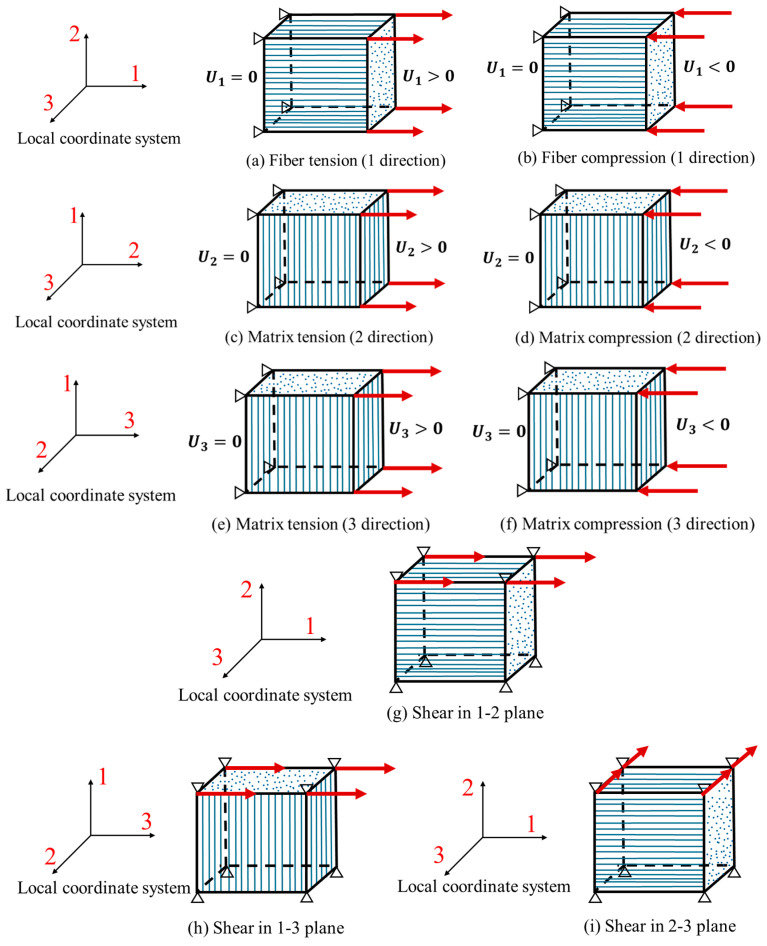
Boundary conditions for single-element tests.

Fiber tensile and compressive failures and matrix tensile and compressive failures constitute the four fundamental failure modes in the UD laminate. Consequently, it is essential to perform individual element validation under each of these failure modes, with the boundary conditions for each case illustrated in Figure 3. Hereafter, unless otherwise specified, 1-direction corresponds to the fiber direction, 2-direction corresponds to the in-plane matrix direction, and 3-direction corresponds to the out-of-plane thickness direction. Additionally, since the tensile and compressive forces applied to the element are either parallel or perpendicular to the fiber direction, the element remains in equilibrium in the transverse directions of the loading direction. Therefore, the degrees of freedom in the two directions perpendicular to the loading direction are not to be constrained. This boundary setting aims to avoid constraining deformation due to Poisson’s ratio during loading, keeping the model in a uniaxial stress state. Given that the VUMAT utilizes the 3D Hashin damage criterion, additional examinations are conducted on the 3D cases for matrix failure in the thickness direction (3-direction) and shear failures in the out-of-plane directions (2–3 plane, 1–3 plane). The results of each case will be discussed in detail in Section 4.1.1.

#### 3.1.2. Non-Monotonic Tensile–Compressive Loading Verification

In numerical simulations, due to nonlinearities of material and contact, the strain state of elements within a structure is often non-monotonic. Therefore, it is necessary to assess the stiffness degradation behavior of elements under non-monotonic loading conditions. Two types of non-monotonic loading simulations are conducted, one in the fiber direction (1-direction) and the other in the matrix direction (2-direction). The loading boundary conditions for the elements are the same as in Section 3.1.1 (Figure 3a–d). The loading sequence is presented in Table 3. Steps 1 and 3 are determined in a way that they cause damage initiation to the elements without leading to ultimate failure, while the final two steps (step 5 and step 6) result in ultimate tensile failure of the element. The results are displayed and discussed in Section 4.1.3.

#### 3.1.3. Mesh Sensitivity Test

The mesh size of composite material elements has a significant impact on the generation and propagation of crack bands [24,42], as well as on the mechanical behavior of the elements themselves. To evaluate whether the damage model can reduce the influence of the element size on numerical simulation results, this study employs the same validation method used by Falzon and Apruzzese [43] and Reinoso et al. [44]. Cubic models with a side length of 1 mm are constructed, containing 1, 27, 125, and 343 three-dimensional solid elements (C3D8R) and two-dimensional continuum shell elements (SC8R), respectively. For simplicity, transverse tension is chosen as the basic loading condition, where a tensile displacement of 0.007 mm in the 2-direction is applied to all models, while the boundary conditions and material properties remain unchanged. The relevant validation results will be discussed in detail in Section 4.1.3.

### 3.2. Characteristic Length Assessment

**Figure 4:**
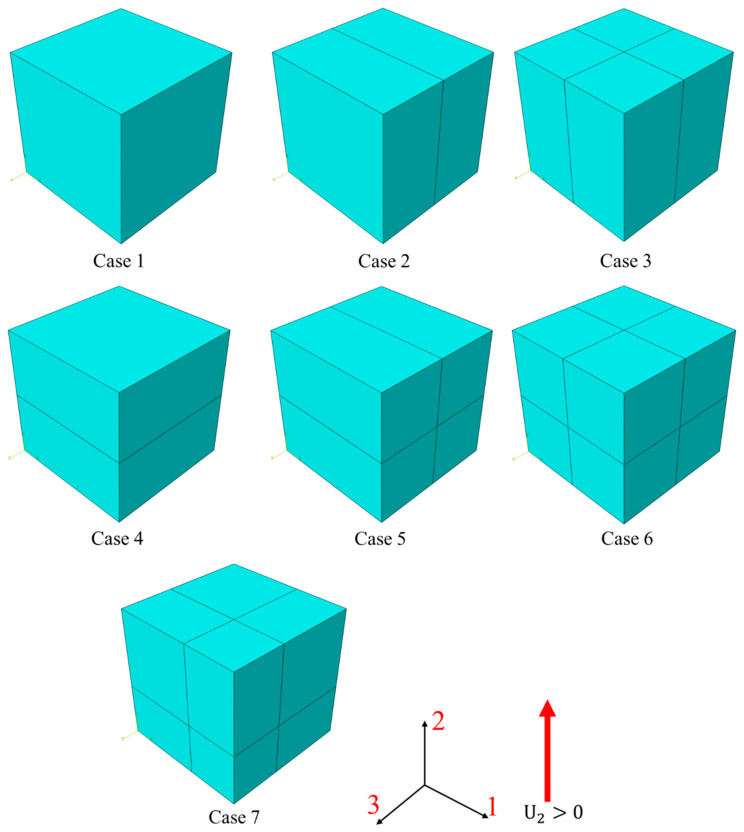
Model with different meshing methods.

Selecting the appropriate characteristic length $L_{c}$ of elements is crucial for obtaining accurate numerical simulation results. This section investigates the impact of $L_{c}$ values on numerical simulation outcomes by performing different mesh divisions on a unit cube model, aiming to determine the optimal characteristic length setting for 3D composite material elements. The different mesh division methods are shown in Figure 4: Cases 1, 2, and 3 and Cases 4, 5, 6 maintain consistent geometric lengths in the loading direction for all internal elements, with the first group containing a single layer of elements and the second group containing two layers. Case 7 also includes two layers of elements but with different geometric lengths in the longitudinal and transverse directions. Simulations are conducted using two approaches to set the $L_{c}$: (a) using the default $L_{c}$ for solid elements in ABAQUS and (b) using a $L_{c}$ based on the actual geometric dimensions of each element in the loading direction. The results of these two approaches are then compared. Similarly, for simplicity, this study focuses on tensile loading in the matrix direction (2-direction), with boundary conditions and material parameters consistent with previous descriptions. The results of this section will be discussed in Section 4.2.

### 3.3. Laminate Level

Following the validation of the VUMAT subroutine at the element level, this section will further investigate whether this PDM can accurately characterize the mechanical response at the laminate scale. The tensile test results on perforated plates presented in [41] are used to compare and validate the numerical simulation results. The material parameters for the IM7/8552 CFRP used in the experiments are obtained from [44], as shown in Table 4.

#### 3.3.1. Tension Test of Single-Ply, Open-Hole Lamina

**Figure 5:**
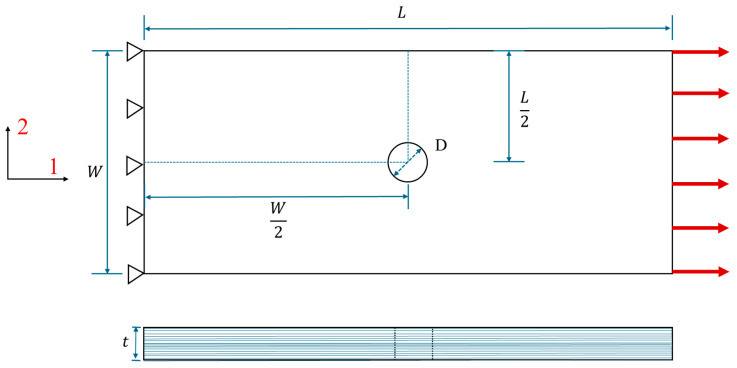
Geometry and boundary conditions of finite element model of open-hole laminate.

To progressively verify the accuracy of this 3D VUMAT subroutine at the laminate scale, three single-ply notched laminas with 0°, 90°, and 45° directions from the multi-ply laminate in the experiment presented in [41] are selected. This preliminary check aims to determine whether it can predict the elastic modulus, ultimate strength, and failure modes of FRP at the single-ply lamina level. The geometric dimensions of each open-hole lamina are 120 mm×12 mm×0.125 mm (*L × W × t*), featuring a central circular hole with a diameter of 2 mm identical to the experimental multi-ply specimen. The dimension diagram can be found in Figure 5. A tensile displacement is applied along the 1-direction without restricting the degrees of freedom of the lamina in the 2-direction.

#### 3.3.2. Tension Test of Open-Hole Laminate [90/0/±45]_3S_

Once the accuracy of the 3D model is validated at both the element and lamina levels, further exploration of its performance within a laminate is undertaken. This section describes the construction of an open-hole plate model, which is developed in accordance with the material parameters and geometric dimensions specified in [41], followed by a tensile simulation test. The single-ply thickness is 0.125 mm, with a layup sequence of [90/0/±45]_3S_. For comparison purposes, five models utilizing the 2D Hashin criterion within ABAQUS’s material library, with an SC8R element type, are also established to compare the results with those of the experimental and 3D numerical results. The geometric layout of each test component can be seen in Figure 5, and the geometric dimensions are listed in Table 5. The interlaminar properties of IM7/8552 [44] are provided in Table 6.

## 4. Results and Discussion

### 4.1. Element Verification

#### 4.1.1. Single-Element Verification

The results of the single-element verification are presented in Table 7, demonstrating that all four failure modes can be accurately characterized. The input parameters and the results obtained from the numerical simulation are in complete agreement. Additionally, it is observed that, apart from shear damage, the evolution of the damage variable is smooth and can be directly correlated with the stress–strain relationship. It is worth noting that for the shear damage mode, when final failure occurs, the shear stiffness ($G_{12}$, $G_{13}$, and $G_{23}$) does not drop to zero, and the element still retains 10% residual shear stiffness. This is because the shear damage coefficient $S_{mt}$ in this subroutine is set to 0.9 (as discussed in Section 2.1).

Thus, it can be confirmed that this PDM is capable of accurately reflecting the mechanical behavior and ultimate failure when a single element is subjected to simple stress conditions.

Additionally, it is worth mentioning that single-element tests using the built-in 2D Hashin material model within ABAQUS were also conducted. While it produced correct results under plane stress conditions (1–2 plane), it was found to be unsuitable when out-of-plane stress components (S33, S23, and S13) were present.

#### 4.1.2. Non-Monotonic Tensile–Compressive Loading Verification

**Figure 6:**
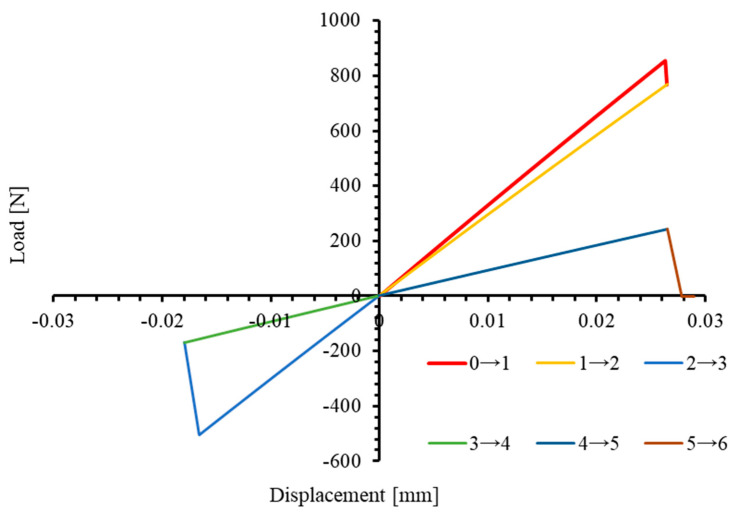
Load−displacement curves of 3D PDM under uniaxial longitudinal (1−direction) tensile–compressive non-monotonic load.

Figure 6 illustrates the force–displacement curve of a solid element utilizing enhanced 3D VUMAT subjected to six stages of uniaxial non-monotonic loading in the fiber direction (1-direction). The first stage is the uniaxial tensile phase (Stage 0→1), where tensile displacement causes stiffness degradation before the end of this stage, resulting in a reduction in $E_{11}$. At point 1, $E_{11}$ decreases from the initial value of 33,457 MPa to approximately 29,150 MPa. As loading continues, the stress state transitions from tension (Stage 0→2) to compression (Stage 2→3). Upon entering the compression phase, the $E_{11}$ remains around 29,150 MPa, indicating that the stiffness degradation induced during the first tensile stage is included in $E_{11}$ under compression. With further compression, $E_{11}$ reduces to approximately 9300 MPa (Stage 3→4). When the element returns to tension after further loading (Stage 4→5), $E_{11}$ remains the same as in Stage 3→4, approximately 9300 MPa. This indicates that the stiffness degradation due to compression is also considered in subsequent tensile loading.

**Figure 7:**
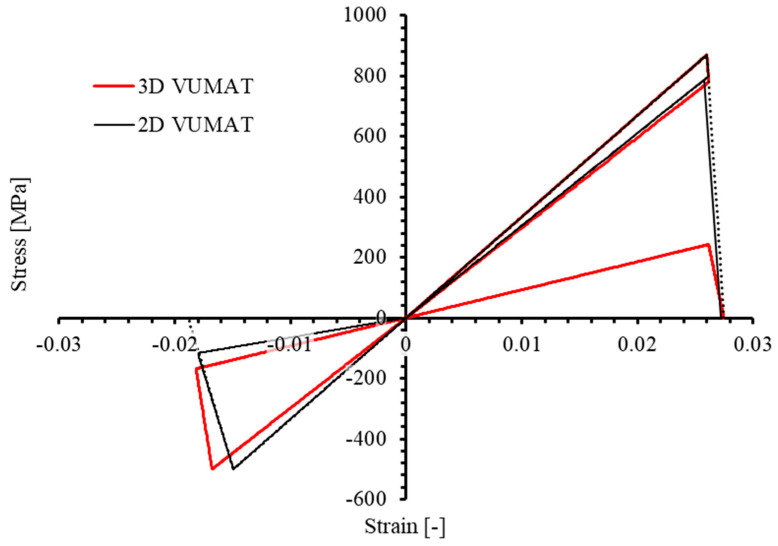
Comparison of stress–strain relationship of 3D PDM and ABAQUS built-in 2D PDM under uniaxial longitudinal (1−direction) tensile−compressive non-monotonic load.

However, the 2D Hashin damage model built-in ABAQUS does not yield the same results. For comparison, Figure 7 shows the stress–strain curves from 2D and 3D PDMs under identical non-monotonic loading. It is evident that the two curves do not align. In the 2D model, stiffness degradation caused by the initial tensile loading in the 1-direction is not considered in the following compression phase: the modulus $E_{11}$ at the end of initial tensile loading is about 29,100 MPa, yet it remains equal to the initial modulus of 33,457 MPa during the subsequent compression phase. Similarly, when transitioning from compression to tension, stiffness degradation due to compression is not accounted for in the tension phase. The dotted line represents the stress-strain curve of the element under the corresponding monotonic uniaxial load until failure.

**Figure 8:**
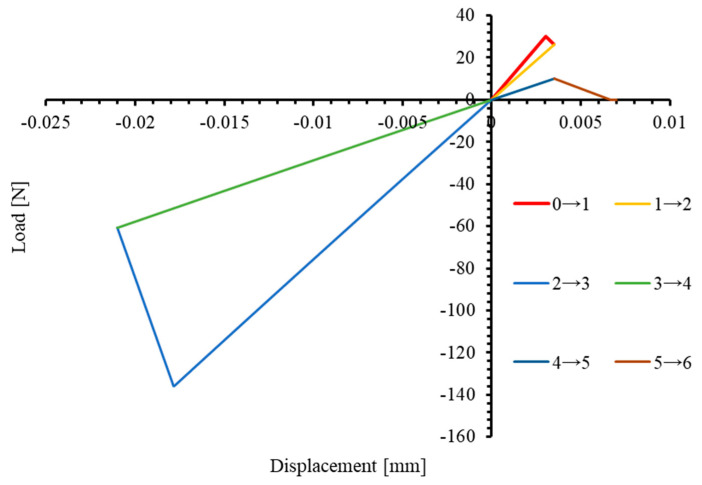
Load–displacement curves of 3D PDM under uniaxial transverse (2-direction) tensile–compressive non-monotonic load.

**Figure 9:**
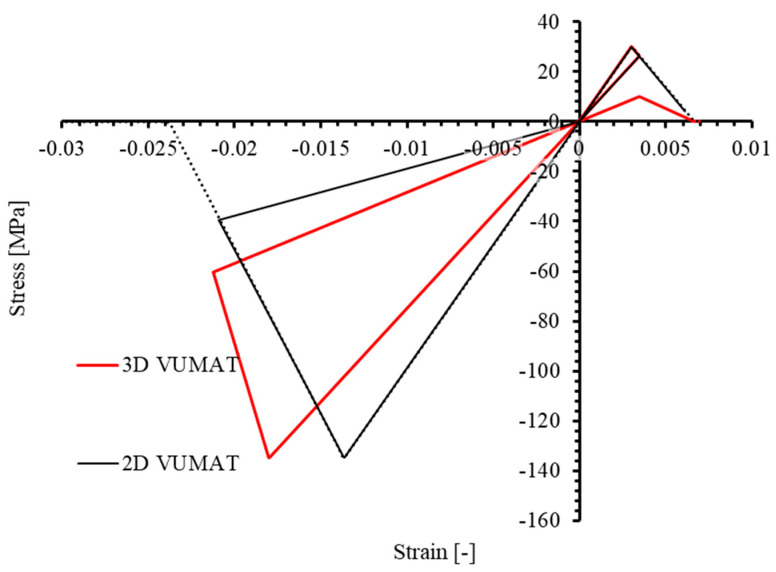
Comparison of stress–strain relationship of 3D PDM and ABAQUS built-in 2D PDM under uniaxial transverse (2-direction) tensile–compressive non-monotonic load.

Similarly, the mechanical behavior of the 3D VUMAT under non-monotonic loading in the transverse direction (2-direction) was characterized, with the force–displacement curve shown in Figure 8. At the end of the first tensile phase (Stage 0→2), degradation of $E_{22}$ due to tension reduces from the initial value of 9883 MPa to approximately 7480 MPa. During the subsequent compression phase (Stage 2→3), $E_{22}$ remains approximately 7480 MPa before any compression damage occurs. When transitioning from the compression phase (Stage 3→4) to the second tensile phase (Stage 4→5), $E_{22}$ remains equal across both stages, approximately 2880 MPa. For comparison, the stress–strain curves of the 3D and 2D models under the same loading conditions are shown in Figure 9. Similar to the previously observed results in the 1-direction, the stiffness degradation caused by tensile and compressive loading is not simultaneously considered in the 2D counterpart.

In conclusion, the 3D PDM developed in this study can effectively characterize the damage behavior of materials under non-monotonic loading by considering tensile and compressive damage in the same direction within a single element. This capability allows for a more accurate characterization of the mechanical behavior of materials subjected to non-monotonic loads. The significance of this attribution lies in its ability to avoid overestimating the stiffness and strength of materials under non-monotonic stress conditions, leading to more reasonable and accurate results [25,44,45,46]. Conversely, models established using the 2D PDM from the ABAQUS material library tend to overestimate the stiffness and strength of materials under non-monotonic loading conditions.

#### 4.1.3. Mesh Sensitivity Test

**Figure 10:**
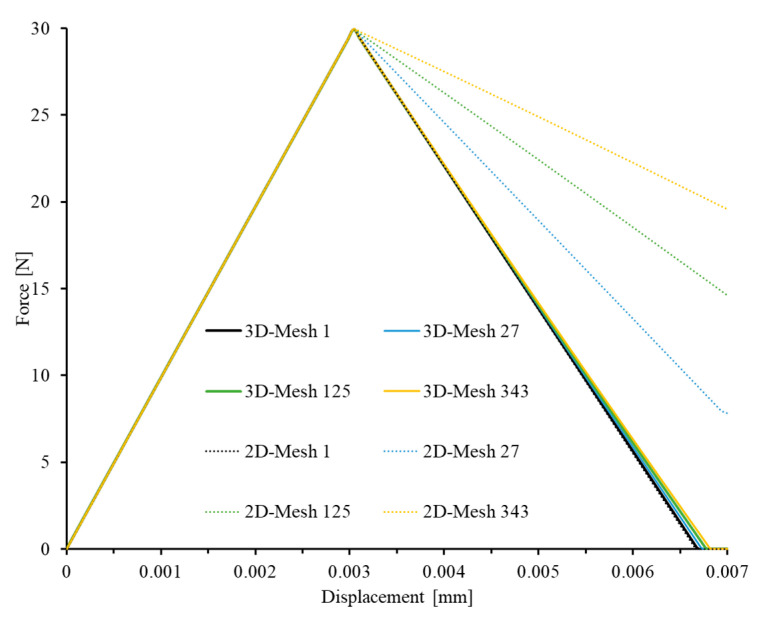
Comparison of load–displacement curves of 3D and 2D elements under uniaxial transverse load (2-direction).

**Figure 11:**
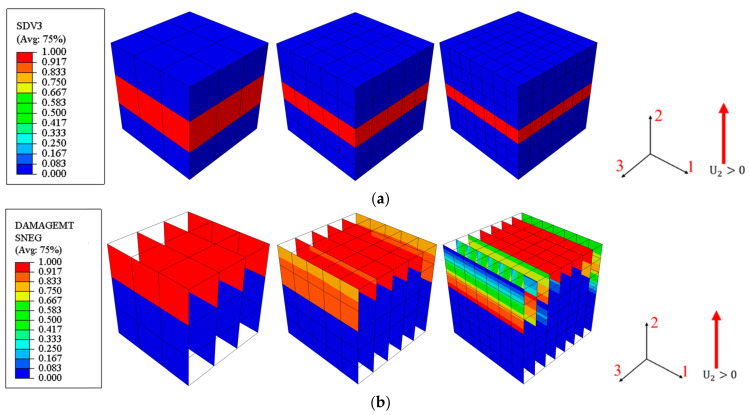
Ultimate failure patterns of (**a**) 3D and (**b**) 2D models under uniaxial tension in in-plane matrix direction (2-direction).

**Figure 12:**
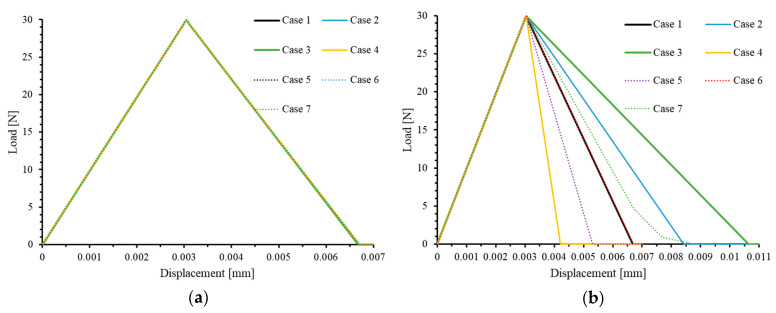
Load–displacement curves of 7 cases under uniaxial tensile load in 2-direction with (**a**) true characteristic length $L_{c}^{d}$ and (**b**) default characteristic length $L_{c}^{t}$.

The results from the mesh sensitivity analysis performed on the developed model are displayed in Figure 10, Figure 11 and Figure 12. The loading direction is transverse to the fibers (in the 2-direction). In Figure 10, it is clear that for the 3D model, the force–displacement curves remain consistent across different mesh densities (Figure 10). Additionally, despite not reducing the strength of the mid-section elements pre-emptively, as described in [44], the crack band still occurs in the middle region, which is perpendicular to the loading direction (Figure 11a, where SDV3 is the matrix tension damage variable). This demonstrates that the model can confine failure to a crack band of one element in width. In contrast, the 2D model cannot achieve the expected results. The rate of stiffness degradation during the damage phase decreases as the mesh density increases (Figure 10), and the failed elements are not concentrated within the same layer’s crack band, as shown in Figure 11b.

These findings confirm that the mechanical behavior of the 3D model is almost unaffected by the mesh size, and the model is capable of developing a reasonable crack band in the central part of the model. This demonstrates the superiority of the presented PDM in ensuring accurate and reliable predictions of failure mechanisms.

### 4.2. Characteristic Length Assessment

In ABAQUS, the default characteristic length ($L_{c}^{d}$) for solid elements is calculated as the cube root of the element’s volume, and the true characteristic length is denoted as $L_{c}^{t}$, which is the actual geometric length of the element in the corresponding direction (2-direction in this test). The ratio of the true characteristic length ($L_{c}^{t})$ of the element in the corresponding direction to its default characteristic length ($L_{c}^{d}$), denoted as γ, is presented in Table 8.

Figure 12a illustrates the tensile load force–displacement curves for seven models using the default characteristic length $L_{c}^{d}$. It is observed that as γ increases, the failure displacement of the models also increases.

This phenomenon is due to the calculation of the element’s fracture using Equation (25):

(25) $$\begin{array}{c} G_{c}=\frac{1}{2}L_{c}\varepsilon \sigma \end{array}$$

When the selected characteristic length ($L_{c}$) exceeds the characteristic length in the loading direction ($L_{c}^{t}$), the element gains a higher fracture energy, resulting in a larger failure displacement. Conversely, if $L_{c}$ is smaller than $L_{c}^{t}$, the element will exhibit a smaller failure displacement. Only when the assigned characteristic length $L_{c}$ is equal to $L_{c}^{t}$ will the element exhibit the expected fracture energy and failure displacement. The true characteristic length of an element ($L_{c}^{t}$) in a given loading direction is defined by its geometric length in that direction.

To validate this conclusion, we assigned the value of $L_{c}^{t}$ as the characteristic length for each element in all seven models. The results are shown in Figure 12b, which demonstrate that, after assigning the correct $L_{c}$ values, all the test cases exhibit accurate load–displacement curves. Additionally, Case 7 further illustrates that even if the geometric lengths of elements in different layers vary in the loading direction, as long as each element is assigned a characteristic length that matches its geometric dimensions, the damage-evolution process of the model can still be accurately represented.

### 4.3. Laminate Level Verification

#### 4.3.1. Tension Test of Open-Hole Single Ply

This section discusses the stress distribution and damage evolution of single 0°, 45°, and 90° notched lamina under tensile loading, respectively.

- Tension test on a 0° open-hole lamina

**Figure 13:**
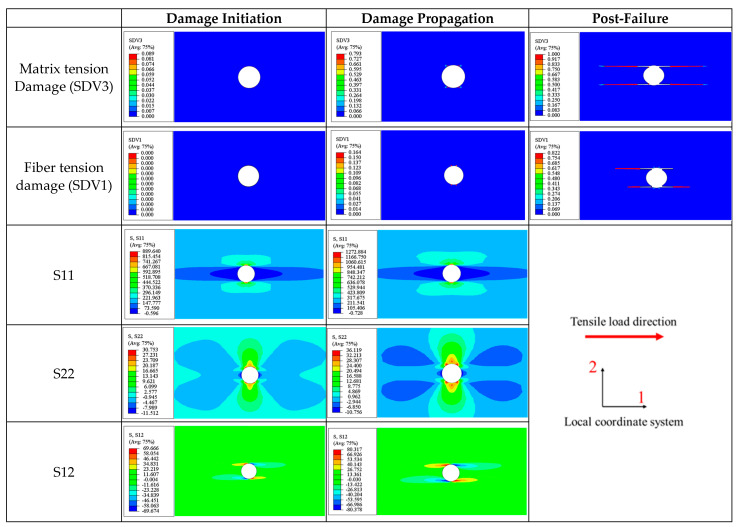
Tensile matrix and fiber damage variables and the distribution of corresponding stress components for 0° lamina during damage evolution.

The initial damage in the lamina is caused by matrix tensile failure (SDV3), with the failure sites located at the edges of the hole and symmetrically distributed along the axis of the plate (see Figure 13). At this stage, no fiber tensile damage is observed. This contrasts with the damage in an unperforated 0° ply under longitudinal tensile load, which is dominated by fiber tensile damage (SDV1). This difference arises because, in the case of a notched lamina, the vicinity of the hole experiences a complex stress state due to stress concentration. According to Figure 13, the distributions of longitudinal normal stress (S11), transverse normal stress (S22), and in-plane shear stress (S12) are observed. These stresses reach their maximum values at the edges of the hole, with respective magnitudes of 889.6 MPa, 30.8 MPa, and 69.7 MPa. However, based on the damage initiation criterion described in Section 2.2, these stress levels are insufficient to initiate fiber damage in the lamina but are adequate to cause matrix tensile failure. Although the maximum value of the transverse normal stress is only 30.8 MPa, the high level of shear stress (69.7 MPa) at this location initiates matrix tensile damage under the combined effect of transverse normal stress (S22) and in-plane shear stress (S12). This further substantiates the rationale behind the enhanced method for calculating the equivalent damage initiation strain proposed in Section 2.5.

As the tensile load increases, the matrix tensile damage continues to evolve, and fiber tensile damage begins to occur at this location as well, although the extent of fiber tensile damage lags behind that of the matrix tensile damage. At this point, the in-plane stress distribution in the lamina is similar to that at the initiation of damage, albeit at elevated stress levels.

As shown in Figure 13, the ultimate failure of the perforated plate is caused by both fiber tensile and matrix tensile failure modes, with cracks aligned parallel to the fibers and the loading direction, which is consistent with the general understanding that cracks in composite materials propagate along the fiber direction. Thus, it is concluded that the failure of the perforated single 0° ply under tension is controlled by both matrix and fiber failures.

Additionally, the stress–strain curve of the 0° ply under tension is shown in Figure 16. The calculated elastic modulus in the tension direction is 168,116.21 MPa, slightly lower than the elastic modulus of 171,420 MPa calculated for an unperforated 0° ply using classical laminate plate theory (CLT). This confirms the accuracy of the single 0° ply during the elastic stage.

- Tension test on a 45° open-hole lamina

**Figure 14:**
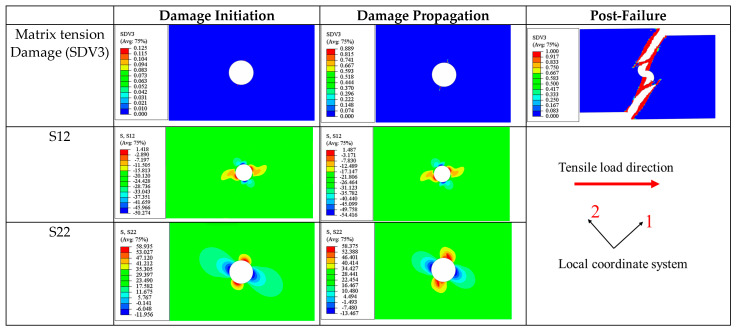
Tensile matrix damage variables and the distribution of transverse normal and in-plane shear stresses for 45° lamina during damage evolution.

For a single 45° open-hole lamina, the most significant stress components during loading are the transverse normal stress (S22) and the in-plane shear stress (S12). And the initial damage is governed by matrix tensile damage (SDV3). The distribution of these two stress components at the onset of damage is depicted in Figure 14. The maximum values of transverse normal stress (S22) and in-plane shear stress (S12) are approximately 58.9 MPa and 50.3 MPa, respectively. Although neither stress component individually reaches the critical value for damage initiation, the combined effect of these stresses can induce matrix tensile failure (Equation (13)), according to Section 2.2. Therefore, matrix tensile damage (SDV3) occurs where S22 is below the critical value (62.3 MPa).

Furthermore, Figure 14 also show the distribution of SDV3 and the stresses S22 and S12 during the damage-evolution process. It can be observed that the cracks propagate along the fiber direction, and the stress distribution remains similar to that at the initial moment of damage.

Lastly, Figure 14 displays the direction of crack propagation at failure for the 45° open-hole lamina, which, as for the 0° lamina, is parallel to the fiber direction. It can also be observed that the two parts of the lamina experience relative displacement in the 2-direction. This is because the degree of freedom in the 2-direction is not constrained during the loading process.

Additionally, the stiffness of the 45° lamina during the elastic phase is validated. By plotting the stress–strain curve during the loading process (Figure 16), the elastic modulus of the 45° lamina in the loading direction is determined to be approximately 13,328.5 MPa, slightly lower than the value of 13,433.6 MPa calculated using classical laminate plate theory (CLT) for an unperforated lamina.

- Tension test on a 90° open-hole lamina

**Figure 15:**
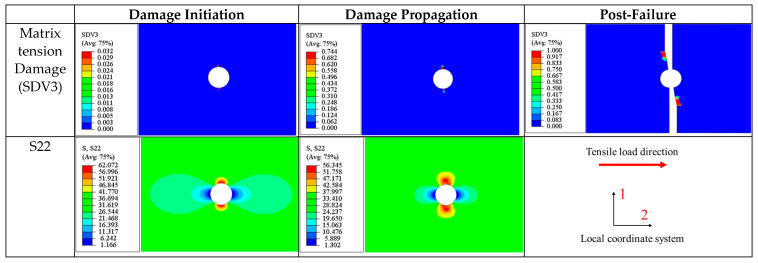
Tensile matrix damage variables and the distribution of transverse normal stress for 90° lamina during damage evolution.

Compared to the 0° and 45° plies, the stress distribution in the 90° lamina under tensile load is relatively simple (Figure 15). Throughout the loading process, the transverse normal stress (S22) is significantly higher than the stresses in other components. Damage initiation is governed by matrix tensile damage (SDV3). The distribution of the transverse stress is depicted in Figure 15, with a maximum value of 62.07 MPa located at the upper and lower edges of the hole.

After damage occurrence, the peak transverse normal stress (S22) of 56.35 MPa is reduced from the initial maximum value (62.07 MPa) due to stiffness reduction. During this process, cracks continue to extend along the fiber direction.

At complete fracture, the cracks also propagate along the fiber direction (perpendicular to the loading direction), which also indicates that the tensile failure of the 90° open-hole lamina is predominantly governed by matrix tensile damage.

**Figure 16:**
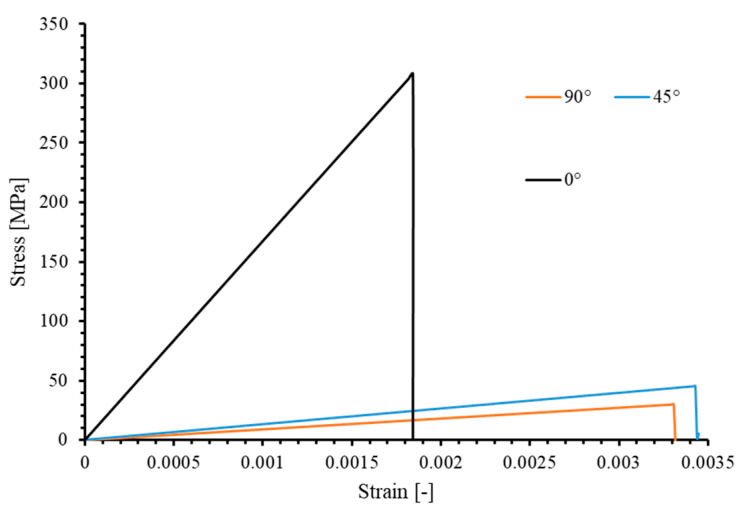
Load–displacement curves of the 0°, 90° and 45° open-hole lamina under tension load.

Similarly, the stress–strain curve under tension (Figure 16) reveals that the elastic modulus of the 90° lamina along the longitudinal direction of the plate is 9011.24 MPa, slightly less than the value of 9080 MPa calculated using Classical Laminate Theory (CLT) for the unperforated condition.

In the tensile testing simulations of the 0°, 45°, and 90° unidirectional open-hole laminas, it is observed that while the damage in the 0° case is governed by both matrix tensile (SDV3) and fiber tensile (SDV1) damages, the failures in the other two cases are always dominated by matrix tensile failure (SDV3). Additionally, the cracks induced by matrix damage always propagate along the fiber direction. These observations are consistent with experimental results by Prabhakar and Waas [47], Khechai et al. [48], and Zhang et al. [34], as well as finite element studies by Prabhakar and Waas [47], Forghani et al. [49], Zhang et al. [34], and Divse et al. [16].

Therefore, it can be concluded that the VUMAT subroutine accurately characterizes the damage evolution and failure modes of 0°, 45°, and 90° open-hole laminas during tension.

#### 4.3.2. Comparison of Numerical Simulation Results in Tension Test of Open-Hole Laminate [90/0/± 45]_3S_ with Experimental Results

To verify the accuracy of the three-dimensional PDM model at the laminate level, this section first conducts an in-depth analysis and discussion of the stress distribution states and final failure modes of each ply in the elastic and softening stages using the 3D VUMAT. Subsequently, it compares the overall ultimate strength and failure modes predicted by the 3D laminate model with the experimental results obtained by Camanho et al. [41]. Furthermore, the ultimate strength of the open-hole laminate obtained using the 2D Hashin criterion from ABAQUS’s material library is also presented. This aims to highlight the superiority of the 3D VUMAT discussed in this paper.

The test specimens consist of five open-hole laminates with varying widths (W) and hole sizes (D), configured in a [90/0/±45]_3S_ layup, maintaining a consistent width-to-diameter ratio (W/D) of 6 for each specimen. The geometric dimensions are provided in Table 5, and the geometric configuration is illustrated in Figure 5.

Initially, a mesh sensitivity analysis is performed on the 3D model with an open-hole size of 2 mm. It is found that the ultimate strength σ increases progressively with decreasing mesh sizes of 1.5 mm, 1.0 mm, 0.75 mm, and 0.5 mm, with respective ultimate strengths of 466.6 MPa, 520.2 MPa, 529.0 MPa, and 537.0 MPa. For the sake of accuracy in the simulation results, a mesh size of 0.5 mm is selected for all subsequent simulations.

Furthermore, to enhance computational efficiency, mass scaling is activated. When the mass scaling factor is set at 100, 500, and 1000, the results remain very close to each other. Therefore, to ensure computational efficiency, the scaling factor is set at 1000.

- Damage prediction for each ply in an open-hole laminate

We first discuss the stress and damage states of different single plies. For simplicity, in this part of the discussion, we chose to analyze in detail a model with a hole size of 6 mm, as it represents a moderate size. Although the laminate consists of 20 individual plies, similar damage modes are observed in plies of the same fiber orientation. Therefore, we selected four plies from the 0° plies, ±45° plies, and 90° plies for a detailed discussion, respectively. Additionally, to clearly demonstrate the damage process of each ply, we select four critical moments to observe the stress distribution and damage state of each ply, which are at a 0.5 ultimate load, 0.8 ultimate load, ultimate load, and post-failure.

(a) 0° ply

Throughout the tensile process, the longitudinal normal stress (S11), transverse normal stress (S22), and in-plane shear stress (S12) are all significant, with damage being predominantly governed by fiber tensile (SDV1) and matrix tensile (SDV3) damage.

**Figure 17:**
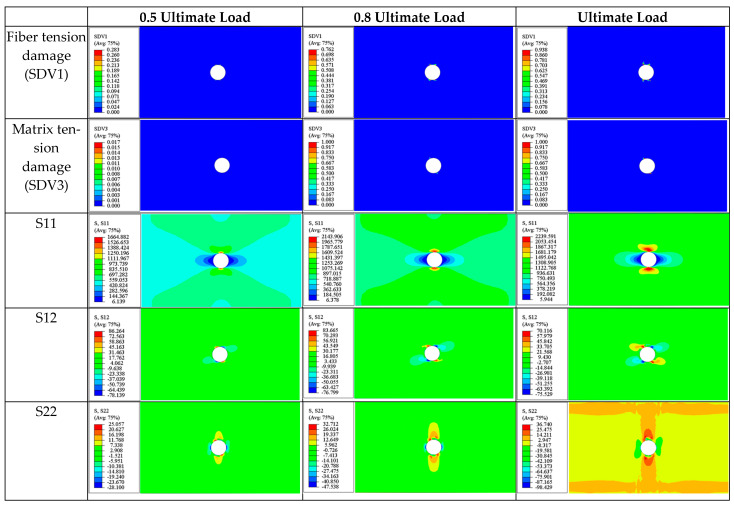
Tensile matrix and fiber damage variables and the distribution of corresponding stress components for 0° plies of [90/0/±45]_3S_ laminate at different load levels.

At the onset of damage (a 0.5 ultimate load), due to the stress concentration, the maximum values of S11, S12, and S22 occur at the edge of the hole (Figure 17), with maximum stress values of 1665 MPa, 86.26 MPa, and 28.1 MPa, respectively. Although these values do not individually reach the ultimate strength for damage, the combined effect of these stresses initiates fiber tensile and matrix tensile damage in this region.

As loading progresses, at a 0.80 ultimate load, both fiber and matrix tensile damage continue to evolve, with the evolution of matrix tensile damage (SDV3) being faster than that of fiber tensile damage (SDV1), reaching 1 in certain areas, indicating matrix tensile failure at these locations (Figure 17). This observation is consistent with phenomena observed in Section 4.3.1 for the single 0° lamina, where the stress distribution at this stage shows little change, with maximum stresses still occurring at the edge of the hole.

Upon reaching the ultimate load, the damage and stress distribution remain similar to the previous stage. At this point, significant stiffness degradation has occurred around the edges of the circular hole, with S11 reaching 2240 MPa at the upper and lower edges of the hole, markedly higher than the stress levels in other plies along the tensile direction. This suggests that most of the load in the laminate tensile test is borne by the 0° ply, with the tensile load primarily supported by this layer.

After failure, the collective tensile damage and fiber tensile damage distribution in the 0° ply are shown in Figure 21. The final fracture occurs in the middle of the plate and passes through the circular hole, with the crack direction perpendicular to the loading direction. Both matrix and fiber tensile damages are approximately 1 at the final fracture, indicating that the final failure is caused by a combination of both damage modes.

The observations in this section are similar to those in Section 4.3.1, suggesting that whether acting alone or within different plies of the laminate, the final failure of the 0° ply is caused by a combination of fiber tensile (SDV1) and matrix tensile (SDV3) damage. Throughout the process, the longitudinal normal stress (S11), transverse normal stress (S22), and in-plane shear stress (S12) are significant, with the longitudinal normal stress near the edge of the hole approaching the critical value before failure.

(b) ±45° Ply

The stress state and damage modes of the ±45° plies are very similar; thus, they are discussed together. In the ±45° laminas during the tensile process, the damage is predominantly controlled by matrix tensile damage (SDV3), while the stress state is dominated by in-plane shear stress (S12) and transverse normal stress (S22).

**Figure 18:**
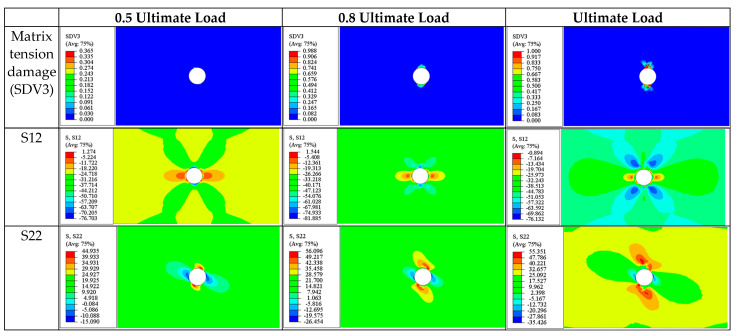
Tensile matrix and fiber damage variables and the distribution of corresponding stress components for 45° plies of [90/0/±45]_3S_ laminate at different load levels.

**Figure 19:**
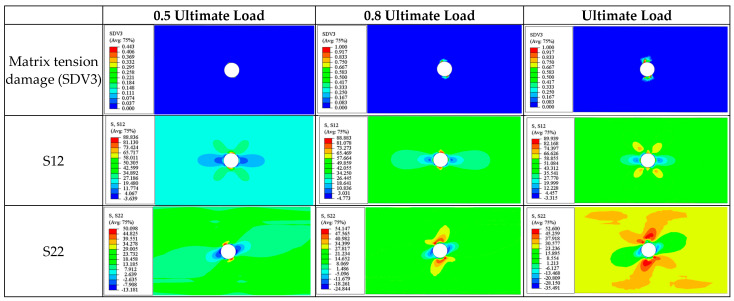
Tensile matrix and fiber damage variables and the distribution of corresponding stress components for −45° plies of [90/0/±45]_3S_ laminate at different load levels.

Taking the 45° ply as an example, at the onset of damage (0.5 ultimate load), S22 and S12 are most significant at the upper and lower edges of the circular hole (Figure 18), measuring 44.93 MPa and 76.6 MPa respectively. Due to the combined effect of the transverse stress S22 and shear stress S12, matrix tensile damage occurs in this area (Figure 18). Similarly, the −45° ply exhibits comparable damage and stress distributions at this moment, as seen in Figure 19.

As the loading continues, the damaged area expands, and the severity of damage evolves (Figure 18 and Figure 19). Additionally, it can be observed that S12 shows a symmetric distribution, while S22 displays an antisymmetric distribution.

At ultimate failure, some areas near the circular hole in the ±45° plies experience matrix tensile failure due to stress concentrations. However, since the ultimate load-bearing capacity of the laminate is primarily provided by the 0° ply, the matrix tensile damage in the ±45° plies has a very limited impact on the overall stiffness of the laminate and does not trigger the final failure of the laminate.

The final failure pattern of the ±45° plies is shown in Figure 21. The fracture zone is still located in the middle of the plate and passes through the hole area perpendicular to the loading direction. Matrix tensile failure also occurs in the triangular areas on both sides of the fracture zone. It can be observed that the cracks in the triangular areas on both sides are not continuous and are distributed intermittently. This phenomenon can be explained by the stress distribution pattern near matrix cracks mentioned in the literature [50], which states that within a certain critical length ($l_{c}^{\prime }$), the closer the area is to the crack, the lower the stress level. Therefore, new cracks can only occur at the boundary or outside of the critical length ($l_{c}^{\prime }$).

(c) 90° Ply

The damage process in the 90° ply is predominantly driven by matrix tensile damage, with the most significant stress components being S22 (transverse normal stress) and S12 (in-plane shear stress).

**Figure 20:**
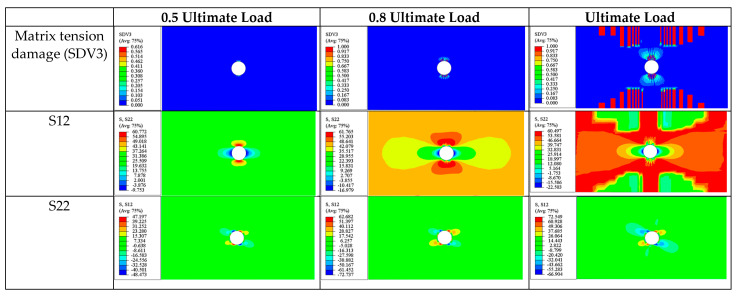
Tensile matrix and fiber damage variables and the distribution of corresponding stress components for 90° plies of a [90/0/±45]_3S_ laminate at different load levels.

At a 0.5 ultimate load, as seen in Figure 20, the maximum transverse normal stress S22 occurs at the areas immediately adjacent to the edges of the hole (60.8 MPa), where collective tensile damage occurs). The maximum shear stress is distributed anti-symmetrically along the centerline of the plate, with a maximum value of 48.5 MPa. This suggests that matrix tensile damage results from the combined effects of these stresses but is dominated by component S22.

As the damage evolves, the stress distribution at a 0.8 ultimate load is similar to that at the onset of damage (0.5 ultimate load). At this stage, several small transverse cracks appear in the upper and lower regions around the hole caused by collective tensile damage (SDV3) and extending laterally across the plate (parallel to the fiber direction).

When the laminate reaches its ultimate strength, isosceles triangular damage zones appear above and below the hole, with the cracks distributed intermittently along the edges of the triangles, similar to the damage pattern discussed in the ±45° ply. Additionally, it can be observed that the stress levels in areas without stiffness degradation are significantly higher than in the damaged areas.

**Figure 21:**
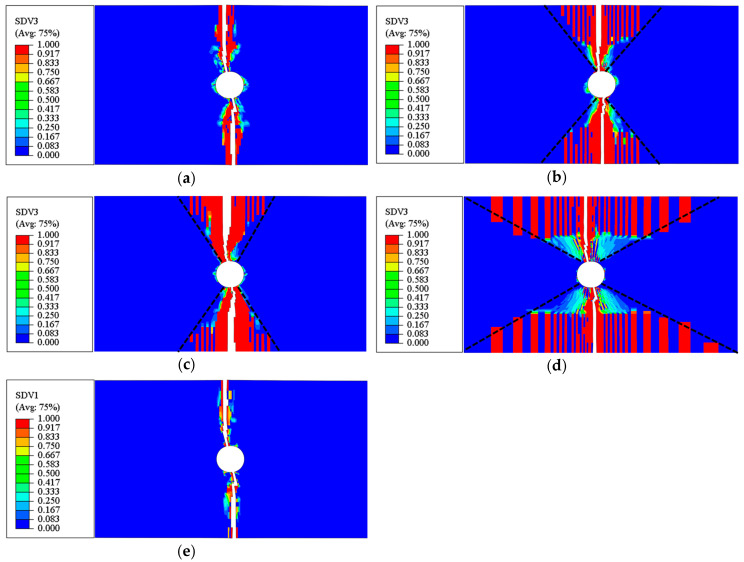
Ultimate failure of each ply of [90/0/±45]_3S_ laminate. (**a**) Ultimate matrix tension damage (SDV3) of 0° ply; (**b**) ultimate matrix tension damage (SDV3) of 45° ply; (**c**) ultimate matrix tension damage (SDV3) of −45° ply; (**d**) ultimate matrix tension damage (SDV3) of 90° ply; (**e**) ultimate fiber tension damage (SDV1) of 0° ply.

The ultimate failure of the 90° ply is also caused by matrix tension damage (SDV3). Similar to the 0° and ±45° plies previously discussed, the final fracture zone at ultimate failure is perpendicular to the loading direction (Figure 21).

In summary, before the final failure, for most of the plies in the [90/0/±45]_3S_ laminate (except for the 90° plies), cracks do not widely propagate but are concentrated around the hole. At final failure, all four ply orientations produce transverse fracture zones passing through the hole and perpendicular to the loading direction. Additionally, except for the 0° ply, triangular damage zones caused by matrix tensile damage appear on both sides along the fracture zone. Apart from the 0° ply, where the ultimate failure is caused by a combination of matrix tensile and fiber tensile damages, the ultimate damage in the other plies with different orientations is predominantly driven by matrix tensile damage (SDV3). The tensile load is primarily borne by the 0° ply, and the final failure of the laminate is caused by the fracture of this ply.

- Laminate overall ultimate load and failure mode

**Figure 22:**
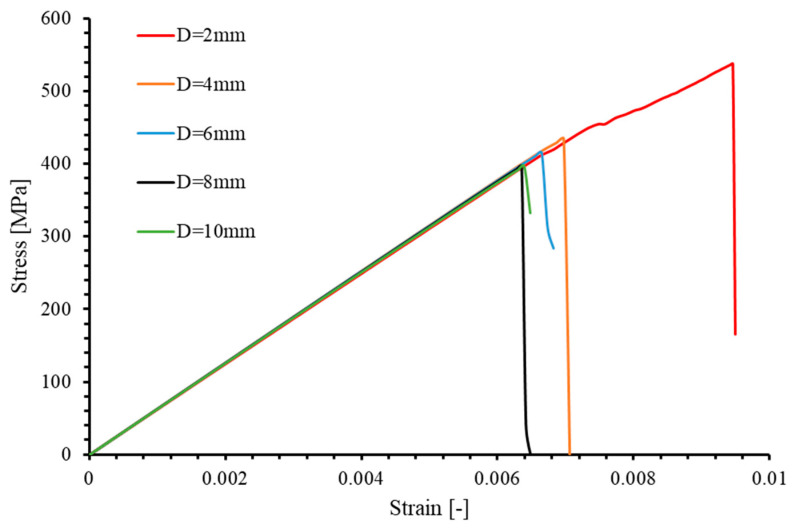
Stress–strain curves predicted by numerical simulation for [90/0/±45]_3S_ laminates with different opening sizes.

The longitudinal stress level σ (MPa) of the laminate is defined as $\left(\sigma =\frac{P}{WT}\right)$, where P (N) is the tensile load, W (mm) is the width, and T (mm) is the total thickness of the laminate. This definition allows for the generation of stress–strain curves for specimens with various hole sizes under tensile loading, as shown in Figure 22.

In the calculations based on Figure 22, it is found that for the specimen with a 6 mm hole diameter, the predicted elastic modulus from the numerical simulation is 62,774.5 MPa, which is slightly lower than the 64,598.8 MPa stiffness calculated using Classical Lamination Theory (CLT) for an unperforated component, with a reasonable error of 2.8%. Additionally, the other four specimens also demonstrated the same elastic modulus.

**Figure 23:**
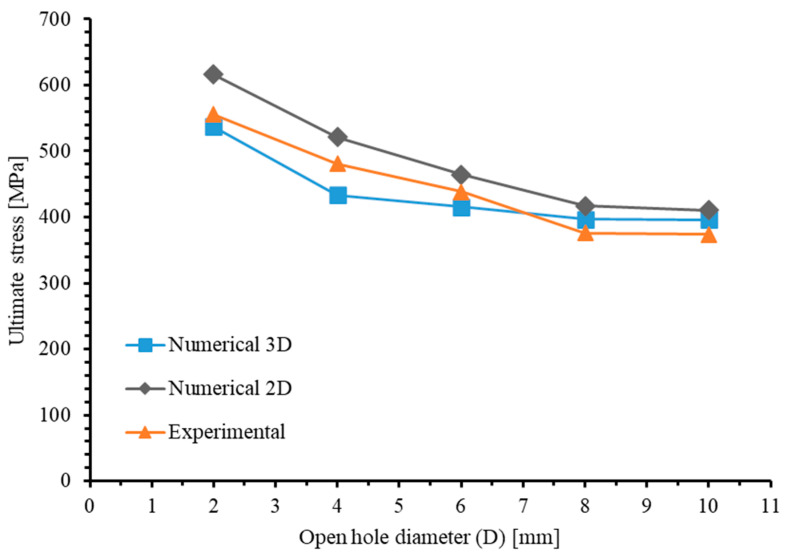
Comparison of size effects from experimental and numerical results.

The failure stresses under tensile loading for the five specimens, as obtained from experimental and numerical simulation results, are listed in Table 9. For the 3D model, the maximum error observed in numerical simulations compared to experimental results is 9.75%, occurring in the specimens with a 4 mm hole diameter, while the minimum error, at −3.37%, is found in the specimens with a 2 mm hole diameter. For the 2D model, the maximum error is 10.89% (D = 8 mm), and the minimum error is 5.88% (D = 6 mm). Figure 23 illustrates the relationship between ultimate stress and open hole diameter (D) in the 2D,3D numerical and experimental results. Despite the discrepancies in the predicted ultimate strengths of the five specimens, the trends in strength variation with respect to laminate size, as obtained from both 3D and 2D models, are entirely consistent with the experimental results. Specifically, as the size of the specimens increases, their ultimate strength monotonically decreases. Both numerical simulations (2D and 3D cases) and experimental findings indicate that the largest difference in ultimate strength is between laminates with hole diameters of 2 mm and 4 mm. Furthermore, the change in ultimate strength decreases with increasing component size, indicating that the size effect diminishes as the size scale increases. When D = 8 mm, the laminate is essentially unaffected by size effects.

However, it is worth noting that the mean absolute error of the predicted ultimate strengths using the 2D model is 9.28%, which is higher than the mean absolute error of 6.00% for the 3D model. Furthermore, the ultimate strength predictions obtained from the 2D model are consistently higher than the experimental values. There are two main reasons for this: (a) the 2D model does not account for out-of-plane stress components, causing the softening phase to occur “later”; (b) the 2D model cannot simultaneously account for tensile and compressive damage in the same direction due to non-monotonic loading, resulting in a model with a higher ultimate bearing capacity (as compared and discussed in Section 4.1.2).

Therefore, compared to the 2D Hashin damage model in ABAQUS’s material library, this 3D PDM, by considering stress components in the thickness direction and stiffness degradation induced by non-monotonic loading, can more accurately predict the ultimate strength and size effect of perforated plates under tensile loads.

**Figure 24:**
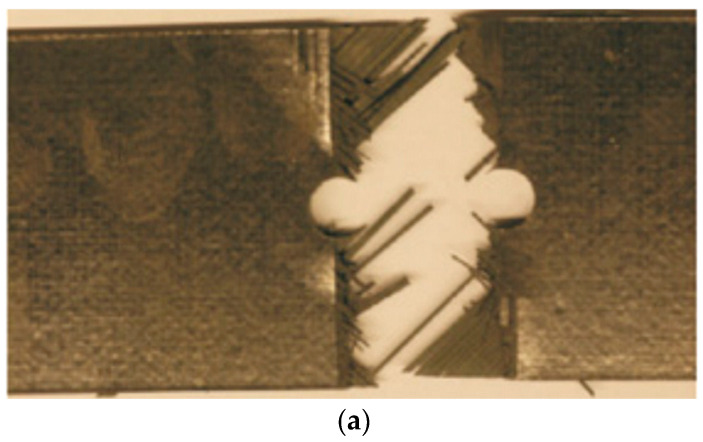

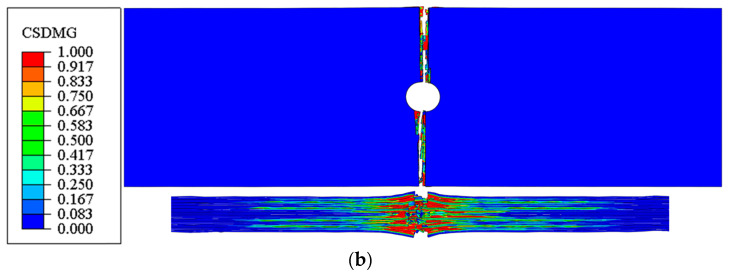
Ultimate failure of [90/0/±45]_3S_ laminate: (**a**) experimental result [41]; (**b**) 3D numerical result.

Furthermore, Figure 24 shows the ultimate failure patterns of the open-hole laminate in both the experiment (Figure 24a) and numerical simulation (Figure 24b). A comparison reveals that both exhibit transverse crack bands running through the central circular hole at the final failure. This indicates that the ultimate failure mode of the open-hole laminate can be reasonably predicted by the 3D PDM presented in this work.

Based on the comparison and discussion of numerical simulation results and experimental findings, it can be concluded that the 3D PDM can accurately predict the mechanical performance and damage mechanisms of notched laminates under tensile loading and reflect the size effects.

## 5. Conclusions

This study integrated a progressive damage model (PDM) based on the 3D Hashin failure criterion in ABAQUS via a VUMAT subroutine and conducted comprehensive validations from the simple single-element level to the complex laminate level. The primary conclusions are as follows.

The subroutine introduces a novel approach for obtaining the equivalent damage initial strain and stress, which more accurately characterizes the mechanical performance and damage evolution of composite materials under complex stress states.

At the single-element scale, the model precisely represents the four failure modes induced by uniaxial stress states: fiber tension damage, fiber compression damage, matrix tension damage, and matrix compression damage. Furthermore, under uniaxial non-monotonic loading, the PDM effectively correlates the damage due to tension and compression in the same direction, yielding reasonable results. At the multi-element scale, the model eliminates the influence of the element size on simulation outcomes, accurately reflecting the mechanical behavior during both the elastic and damage-evolution phases.

The characteristic length significantly influences the accurate representation of the mechanical behavior of composites. For three-dimensional solid elements, the characteristic length should equal the geometric length of the element in the direction corresponding to the damage mode. Choosing the correct characteristic length can eliminate the impact of the mesh size on simulation results.

The model also achieves the expected results at the laminate scale, accurately predicting the mechanical behavior of 0°, ±45°, and 90° single-ply laminates. The cracks in all ply orientations at the final failure align with the fiber direction. The final failure of the 90° and ±45° plies is primarily due to matrix tension failure, while the 0° ply fails due to combined matrix and fiber tension failures.

The enhanced 3D PDM can accurately predict the mechanical behavior and final failure modes of open-hole laminates with varying sizes arranged in a [90/0/±45]_3S_ layup under tensile loading. The average error between the ultimate strength simulated by the 3D model (6.00%) is smaller than the error of the 2D model (9.28%) using the 2D Hashin damage criterion built-in ABAQUS. Additionally, this 3D model is also capable of accurately reflecting the size effect of open-hole laminates, whereby the size effect gradually diminishes as the size of the laminate increases. Observations of the overall failure modes reveal that crack propagation prior to failure is minimal, with no visible cracks in the plies away from the hole, except in the 90° ply. During loading, the 0° ply predominantly bore the tensile load, and its failure ultimately led to the laminate’s failure. At final failure, all plies exhibited a transverse fracture band perpendicular to the loading direction, with approximately triangular damage zones forming on both sides of this band, consistent with experimental observations.

Compared with the built-in 2D model from ABAQUS’s material library, the 3D PDM proposed in this paper allows for a more accurate and realistic representation of the mechanical behavior and damage mechanisms of FRP materials under spatial stress states. However, due to space constraints, this study does not apply the 3D PDM model to scenarios such as low-velocity impacts under spatial loading conditions. In future work, the 3D PDM will be applied to a broader range of spatial mechanical scenarios, such as impact damage and simulations of composites with spatial configurations, in order to further verify and refine the accuracy and superiority of this PDM. It is expected to play a significant role in subsequent finite element simulations of composite materials.

## Figures and Tables

**Table 1:** Equivalent displacement $\delta_{eq}^{i}$ and equivalent stress $\sigma_{eq}^{i}$ for each failure mode.

Failure Mode	$\mathrm{Equivalent}\;\mathrm{Displacement}\;(\delta_{eq}^{i})$	$\mathrm{Equivalent}\;\mathrm{Stress}\;(\sigma_{eq}^{i})$
FT	$L_{c}\sqrt{{\left\langle \varepsilon_{11}\right\rangle }^{2}+{\varepsilon_{12}}^{2}+{\varepsilon_{13}}^{2}}$	$\frac{L_{c}(\left\langle \sigma_{11}\right\rangle \left\langle \varepsilon_{11}\right\rangle +\sigma_{12}\varepsilon_{12}+\sigma_{13}\varepsilon_{13})}{\delta_{eq}^{ft}}$
FC	$L_{c}\left\langle -\varepsilon_{11}\right\rangle$	$\frac{L_{c}\left\langle -\sigma_{11}\right\rangle \left\langle -\varepsilon_{11}\right\rangle }{\delta_{eq}^{fc}}$
MT	$L_{c}\sqrt{{\left\langle \varepsilon_{22}\right\rangle }^{2}+{\left\langle \varepsilon_{33}\right\rangle }^{2}+{\varepsilon_{12}}^{2}+{\varepsilon_{23}}^{2}+{\varepsilon_{13}}^{2}}$	$\frac{L_{c}\left(\left\langle \sigma_{22}\right\rangle \left\langle \varepsilon_{22}\right\rangle +\left\langle \sigma_{33}\right\rangle \left\langle \varepsilon_{33}\right\rangle +\sigma_{12}\varepsilon_{12}+\sigma_{23}\varepsilon_{23}+\sigma_{13}\varepsilon_{13}\right)}{\delta_{eq}^{mt}}$
MC	$L_{c}\sqrt{{\left\langle -\varepsilon_{22}\right\rangle }^{2}+{\left\langle -\varepsilon_{33}\right\rangle }^{2}+{\varepsilon_{12}}^{2}+{\varepsilon_{23}}^{2}+{\varepsilon_{13}}^{2}}$	$\frac{L_{c}\left(\left\langle -\sigma_{22}\right\rangle \left\langle {-\varepsilon }_{22}\right\rangle +\left\langle -\sigma_{33}\right\rangle \left\langle -\varepsilon_{33}\right\rangle +\sigma_{12}\varepsilon_{12}+\sigma_{23}\varepsilon_{23}+\sigma_{13}\varepsilon_{13}\right)}{\delta_{eq}^{mc}}$

**Table 2:** Material properties of E-glass/Polyester.

$E_{11}$ **(MPa)**	$E_{22}=E_{33}$ **(MPa)**	$G_{12}=G_{13}$ **(MPa)**	$G_{23}$ **(MPa)**
33,457	9883	2963	3530
$\upsilon_{12}=\upsilon_{13}$	$\upsilon_{23}$		
0.32	0.4		
$X_{T}$ **(MPa)**	$X_{C}$ **(MPa)**	$Y_{T}$ **(MPa)**	$Y_{C}$ **(MPa)**
870	500	30	135
$S_{12}=S_{13}=S_{23}$ **(MPa)**			
60			
$G_{c}^{ft}$ **(N/mm)**	$G_{c}^{fc}$ **(N/mm)**	$G_{c}^{mt}$ **(N/mm)**	$G_{c}^{mc}$ **(N/mm)**
12	4.7	0.1	1.6

**Table 3:** Loading sequence for non-monotonic benchmark application in longitudinal and transverse directions.

Load Step	$\mathrm{Longitudinal}\;\mathrm{Prescribed}\;\mathrm{Displacement}\;u_{1}$ [mm]	$\mathrm{Transverse}\;\mathrm{Prescribed}\;\mathrm{Displacement}\;u_{2}$ [mm]
1	0.0265	0.0035
2	0.0000	0.0000
3	−0.0180	−0.0210
4	0.0000	0.0000
5	0.0265	0.0035
6	0.0280	0.0068

**Table 4:** Material properties for IM7/8552 [44].

$E_{11}$ **(MPa)**	$E_{22}=E_{33}$ **(MPa)**	$G_{12}=G_{13}$ **(MPa)**	$G_{23}$ **(MPa)**
171,420	9080	5290	3789.5
$\upsilon_{12}=\upsilon_{13}$	$\upsilon_{23}$		
0.32	0.42		
$X_{T}$ **(MPa)**	$X_{C}$ **(MPa)**	$Y_{T}$ **(MPa)**	$Y_{C}$ **(MPa)**
2323.5	1200.1	62.3	199.8
$S_{12}=S_{13}=S_{23}$ **(MPa)**			
92.3			
$G_{c}^{ft}$ **(N/mm)**	$G_{c}^{fc}$ **(N/mm)**	$G_{c}^{mt}$ **(N/mm)**	$G_{c}^{mc}$ **(N/mm)**
81.5	106.3	0.2774	1.31

**Table 5:** Geometric dimensions in laminate test [41].

Laminate	D (mm)	W (mm)	L (mm)	W/D	Total Thickness (mm)
1	2	12	120	6	3
2	4	24	120	6	3
3	6	36	120	6	3
4	8	48	120	6	3
5	10	60	120	6	3

**Table 6:** Interlaminar properties for IM7/8552 [44].

$t_{n}^{\prime }$ (MPa)	$t_{s}^{\prime }=t_{t}^{\prime }$ (MPa)	$G_{1}^{c}$ (N/mm)	$G_{2}^{c}$ (N/mm)	η
33.5141	56.2941	0.28	0.79	1.45

**Table 7:** Stress–strain relationship and evolution of damage variables in single-element verification.

Damage Mode	Stress–Strain Relationship	Evolution of Damage Variables
Fiber tension damage (1−direction)	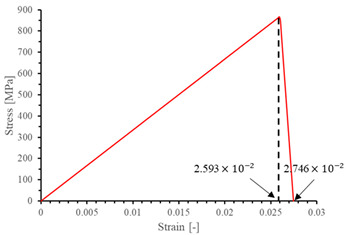	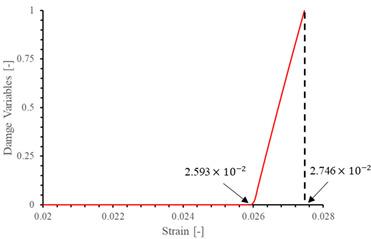
Fiber compression damage (1−direction)	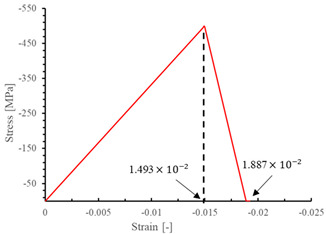	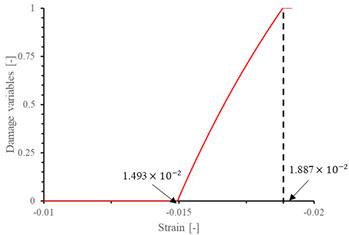
Matrix tension damage (2−direction, 3−direction)	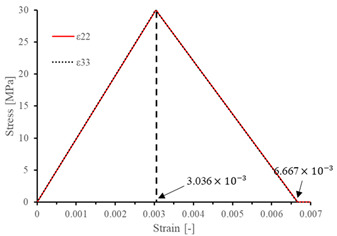	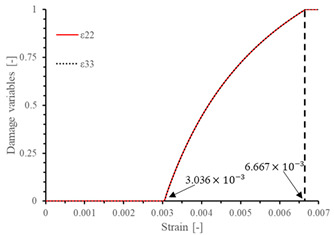
Matrix compression damage (2−direction, 3−direction)	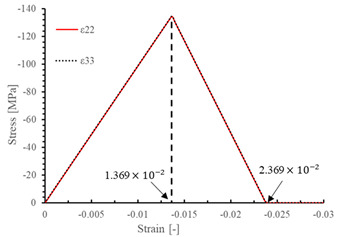	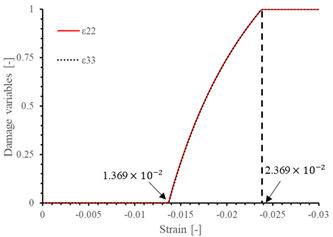
Shear damage (1−2 plane, 1−3 plane, 2−3 plane)	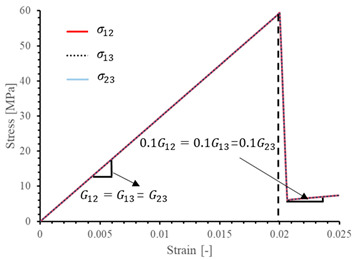	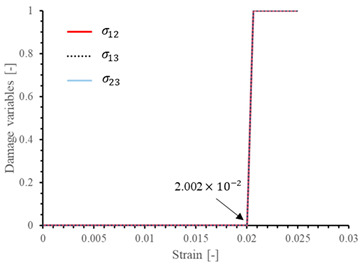

**Table 8:** $L_{c}^{d}$, $L_{c}^{t}$ and γ of different cases.

Case	Element Volume V (mm^2^)	$L_{c}^{d}$	$L_{c}^{t}$	$\gamma \;(L_{c}^{t}/L_{c}^{d}$)
1	1	1	1	1
2	0.5	0.794	1	1.259
3	0.25	0.630	1	1.587
4	0.5	0.794	0.5	0.630
5	0.25	0.630	0.5	0.794
6	0.125	0.5	0.5	1
7	-	-	-	-

**Table 9:** Comparison between experimental and numerical failure stresses (MPa).

Hole Diameter (mm)	$\sigma_{e}$, Experimental	$\sigma_{n}^{3D}$, Numerical	Error (%)	$\sigma_{n}^{2D}$	Error (%)
2	555.7	537.0	−3.37	616.1	+10.87
4	480.6	433.7	−9.75	520.9	+8.39
6	438.7	415.2	−5.36	464.5	+5.88
8	375.7	396.4	+5.51	416.6	+10.89
10	373.7	396.3	+6.05	410.5	+9.85

## Data Availability

The data that support the findings of this study are not publicly available due to privacy and confidentiality agreements. Data are available from the corresponding author upon reasonable request.
